# Network Analysis and Transcriptome Profiling Identify Autophagic and Mitochondrial Dysfunctions in SARS-CoV-2 Infection

**DOI:** 10.3389/fgene.2021.599261

**Published:** 2021-03-16

**Authors:** Komudi Singh, Yun-Ching Chen, Shahin Hassanzadeh, Kim Han, Jennifer T. Judy, Fayaz Seifuddin, Ilker Tunc, Michael N. Sack, Mehdi Pirooznia

**Affiliations:** ^1^Bioinformatics and Computational Biology Laboratory, National Heart, Lung, and Blood Institute, National Institutes of Health, Bethesda, MD, United States; ^2^Laboratory of Mitochondrial Biology and Metabolism, National Heart, Lung, and Blood Institute, National Institutes of Health, Bethesda, MD, United States

**Keywords:** COVID-19, SARS-CoV-2, transcriptomics, co-expression network analysis, inflammation

## Abstract

Analyzing host cells' transcriptional response to severe acute respiratory syndrome coronavirus 2 (SARS-CoV-2) infection will help delineate biological processes underlying viral pathogenesis. First, analysis of expression profiles of lung cell lines A549 and Calu3 revealed upregulation of antiviral interferon signaling genes in response to all three SARS-CoV-2, MERS-CoV, or influenza A virus (IAV) infections. However, perturbations in expression of genes involved in inflammatory, mitochondrial, and autophagy processes were specifically observed in SARS-CoV-2-infected cells. Next, a validation study in infected human nasopharyngeal samples also revealed perturbations in autophagy and mitochondrial processes. Specifically, mTOR expression, mitochondrial ribosomal, mitochondrial complex I, lysosome acidification, and mitochondrial fission promoting genes were concurrently downregulated in both infected cell lines and human samples. SARS-CoV-2 infection impeded autophagic flux either by upregulating GSK3B in lung cell lines or by downregulating autophagy genes, SNAP29, and lysosome acidification genes in human samples, contributing to increased viral replication. Therefore, drugs targeting lysosome acidification or autophagic flux could be tested as intervention strategies. Finally, age-stratified SARS-CoV-2-positive human data revealed impaired upregulation of chemokines, interferon-stimulated genes, and tripartite motif genes that are critical for antiviral signaling. Together, this analysis has revealed specific aspects of autophagic and mitochondrial function that are uniquely perturbed in SARS-CoV-2-infected host cells.

## Introduction

Severe acute respiratory syndrome coronavirus 2 (SARS-CoV-2) is a beta-coronavirus and the cause of the Coronavirus Disease 2019 (COVID-19) pandemic. Lack of effective treatment strategy and vaccine makes SARS-CoV-2 infection a big threat to human health and well-being. Understanding the cellular processes impacted in the host cells responding to a virus infection will be necessary to understand the virus pathogenesis and to develop drug intervention strategies.

COVID-19 presents as a wide range of clinical manifestations, ranging from asymptomatic to respiratory failure or multiorgan and systemic manifestations (Cascella et al., 2020; Ludwig and Zarbock, 2020; Wang C. et al., 2020; Zhu et al., 2020). This viral pneumonia outbreak caused by SARS-CoV-2 was first identified in Wuhan, China, in December 2019 (Chan et al., 2020). Since then, the virus has continued to spread globally, with a current transmissibility estimate (R_0_) between 3 and 4 (Fung et al., 2020; Yuen et al., 2020). Several drugs are currently under various phases of clinical trials, and management strategies include supportive medical care for existing cases and social distancing for prevention. Understanding this novel pathogen and the host response it elicits is crucial to combatting the emerging threat to public health.

SARS-CoV-2 is the 7th and most recent addition to human coronaviruses (hCoVs), which include four globally endemic hCoVs that cause a substantial portion of upper respiratory infections (229E, OC43, HKU1, and NL63), as well as two other highly pathogenic strains that have also caused recent pandemics [SARS-CoV and MERS-CoV (Fung et al., 2020; Raoult et al., 2020) in 2002–2003 and 2012, respectively (De Wit et al., 2016)]. All seven hCoVs are single-stranded, positive-sense RNA viruses (Ludwig and Zarbock, 2020). They all have zoonotic origins, with bats as the evolutionary reservoir host of five viruses (229E, NL63, SARS-CoV, MERS-CoV, and SARS-CoV-2). Although SARS-CoV-2 is phylogenetically similar to both MERS-CoV, and SARS-CoV (Wu et al., 2020), there are biological differences. Notably, although SARS-CoV-2 has a lower, but yet undetermined mortality rate, it is distinctly more contagious than these other highly pathogenic hCoVs, causing vastly different epidemiological dynamics.

The hCoVs differentially infect the human respiratory tract. The low pathogenic hCoVs infect the upper respiratory tract, and the highly pathogenic hCoVs infect the lower respiratory tract (Channappanavar and Perlman, 2017). Consistent with this, SARS-CoV, SARS-CoV-2, and MERS-CoV were shown to differentially infect the lung alveolar cell subtypes in cynomolgus macaques (Rockx et al., 2020) and SARS-CoV elicited distinct immune response in different tissues (To et al., 2004). Furthermore, cell tropism study of the SARS-CoV and SARS-CoV-2 in different cell type cultures could partially explain the symptomatic differences of these two virus infections (Chu et al., 2020). Single cell (sc) transcriptomic data of the COVID-19 lung tissue have been analyzed to identify the subset of cells most prone to the SARS-CoV-2 infection and the marker genes associated with the infected cells. One such study intriguingly identified upregulation of the receptor-angiotensin-converting enzyme 2 (ACE2) in the SARS-CoV-2-infected type II pneumocyte population of the lung cells as a potential mechanism facilitating virus infection (Ziegler et al., 2020). Several studies have linked the expression of ACE2 and TMPRSS2 with increased susceptibility to viral entry (Hoffmann et al., 2020; Walls et al., 2020; Yan et al., 2020). In addition to ACE2 and TMPRSS2, another study has shown that the neuropilin-1 (NRP1) could act as a host cofactor and facilitate viral entry (Daly et al., 2020).

One of the intriguing aspects of COVID-19 is that the infection leads to a wide range of the symptoms. The infected patients may be asymptomatic or may present mild to severe symptoms, which likely arise from altered immune response (Garcia, 2020). A dysregulated immune response is caused by rapid viral replication, cytokine storms, delayed interferon response, and macrophage infiltration and excessive proinflammatory cytokines (Channappanavar and Perlman, 2017; Garcia, 2020). This immunopathogenesis mechanism is supported by the observation of decreased viral loads occurring with increased disease severity (De Wit et al., 2016). Severity of illness for SARS-CoV-2 infections is likely impacted by both the direct cytotoxic effects of the virus, and the effectiveness of the complex host response (Mar et al., 2018; Astuti and Ysrafil, 2020; Chen G. et al., 2020). However, efforts to understand the molecular mechanisms driving different clinical outcomes require further study to help develop appropriate drug intervention strategies.

**Figure 1:**
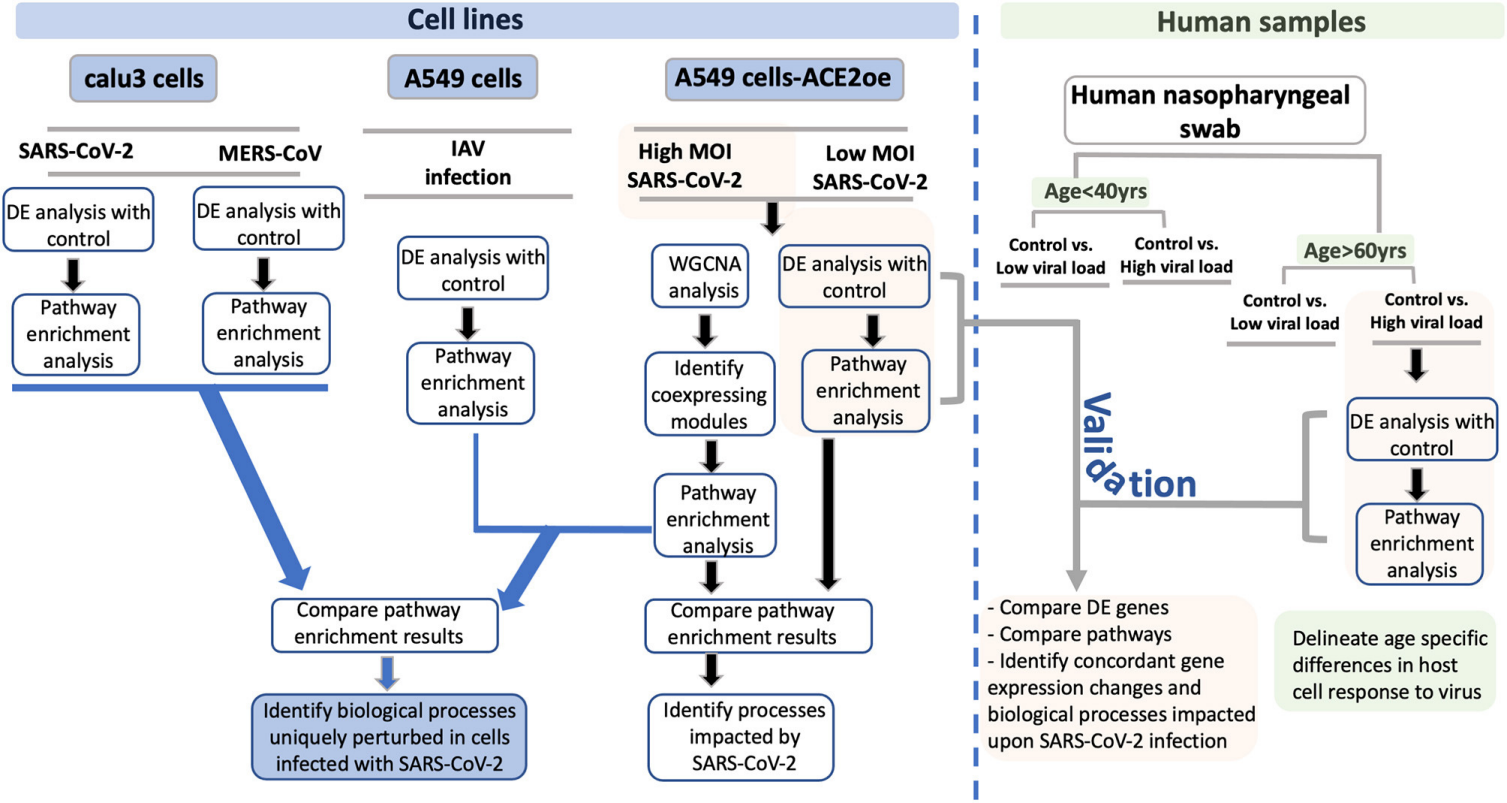
Study schema. A schema highlighting the various datasets used in the study and the downstream analysis performed for each dataset is shown as a flow chart. The datasets utilized in this study are divided into two groups: Blue boxes highlight the cell lines used in the study. The viruses used for infection are indicated between gray horizontal lines. The green box represents the human nasopharyngeal datasets and the age and viral load criteria used to classify these samples. For each pair of infected and control samples, DE analysis was performed to identify DE genes and pathway enrichment analysis was performed to reveal the biological processes to which the DE genes annotated to (shown in the flow chart). From the cell line data, the DE genes and pathway enrichment results were compared to identify BPs that were either uniquely perturbed in SARS-CoV-2 infection or were commonly perturbed in all viral infection (blue lines with arrowhead leading to the blue box). For validating the findings from the cell line data, the DE results from infected human nasopharyngeal samples were analyzed. Specifically, the DE results from the A549 dataset (highlighted in orange box) was compared with the DE results from old age control vs. high viral load positive human nasopharyngeal samples (highlighted in orange box). This comparison will help identify concordant gene expression changes and BPs impacted in both datasets (gray line with arrowhead leading to orange box). Finally, using the age-stratified human nasopharyngeal datasets, age-specific gene expression changes were delineated (green boxes). DE, differential expression; BPs, biological processes; MOI, multiplicity of infection.

To delineate the host cell transcriptional response to the viral infection and potentially identify genes and biological processes (BPs) specifically impacted by SARS-CoV-2 infection, we have utilized gene expression information from several datasets from cell lines (Blanco-Melo et al., 2020), and from human nasopharyngeal samples (Lieberman et al., 2020) classified into young and old groups that were positive for high or low viral loads (see Figure 1, study schema). To facilitate SARS-CoV-2 viral entry, A549 cells transduced with human ACE2 (hACE2) infected with SARS-CoV-2 were utilized (see Methods). The transformed A549 cells and Calu3 cells both revealed viral reads when infected with SARS-CoV-2 (Blanco-Melo et al., 2020). Viral infection in the transduced A549 cells and Calu3 cells presented in this dataset was confirmed by evaluating the percent reads that aligned with the viral genome for each of the infected samples and has been published (Blanco-Melo et al., 2020). Analysis of gene expression profiles of cells infected with either SARS-CoV-2, MERS-CoV, or influenza A virus (IAV) comparisons revealed upregulation of interferon signaling genes. However, the SARS-CoV-2 infection uniquely elicited differential expression of genes involved in inflammation, autophagy, and mitochondrial processes. To validate the findings from cell lines, expression profile of the SARS-CoV-2-positive human nasopharyngeal samples was analyzed. Consistent with the cell line data, the differentially expressed (DE) genes from control vs. infected human samples also annotated to inflammation, autophagy, and mitochondrial processes. Notably, mTOR, mitochondrial ribosomal, mitochondrial complex I, lysosome acidification genes, and mitochondrial fission promoting genes were downregulated in both cell line and human datasets. Perturbation of the mitochondrial function and autophagy could negatively impact the host cells' immune response against the viral infection leading to systemic inflammation (Won et al., 2015; Jang et al., 2019). Decreased expression of mitochondrial fission promoting genes may contribute to hyperfused mitochondria and impaired interferon response (Barbier et al., 2017; Das and Chakrabarti, 2020). In the cell lines, the autophagy flux impeding GSK3B was upregulated. In the infected human samples, several autophagy genes, p62 and SNAP29, were downregulated. Together, these gene expression changes support the idea that the autophagic flux is likely decreased in SARS-CoV-2-infected cells, which may contribute to viral propagation. Therefore, drugs increasing autophagic flux, or lysosome acidification, could be tested as treatment strategies. Furthermore, the gene expression profile of A549 cell line strongly correlated with the lung epithelial lineage basal and ionocyte cell types from the lung single-cell (sc) RNA-seq data. This correlation suggests that the gene expression profile of A549 cells likely reflects lung cells' response to the SARS-CoV-2 infection. Using the age-stratified human nasopharyngeal expression data, we have also delineated some age-specific changes in antiviral signaling, which may provide more insight into the age-dependent differences in viral pathogenicity. Therefore, from this analysis, we have identified some key aspects of autophagy and mitochondrial processes that are uniquely impacted in SARS-CoV-2 infection and are likely representative of host cells' response to the infection.

## Results

To perform an in-depth analysis of the host cells' transcriptional response to SARS-CoV-2 infection, several gene expression datasets from different cell lines and human samples were used. An overview of the datasets analyzed and compared are depicted in Figure 1 (study schema).

### Interferon Autophagy and Mitochondrial Processes Are Impacted in A549 Cells Infected With SARS-CoV-2

#### SARS-CoV-2 (High Viral Titer) vs. Mock

**Figure 2:**
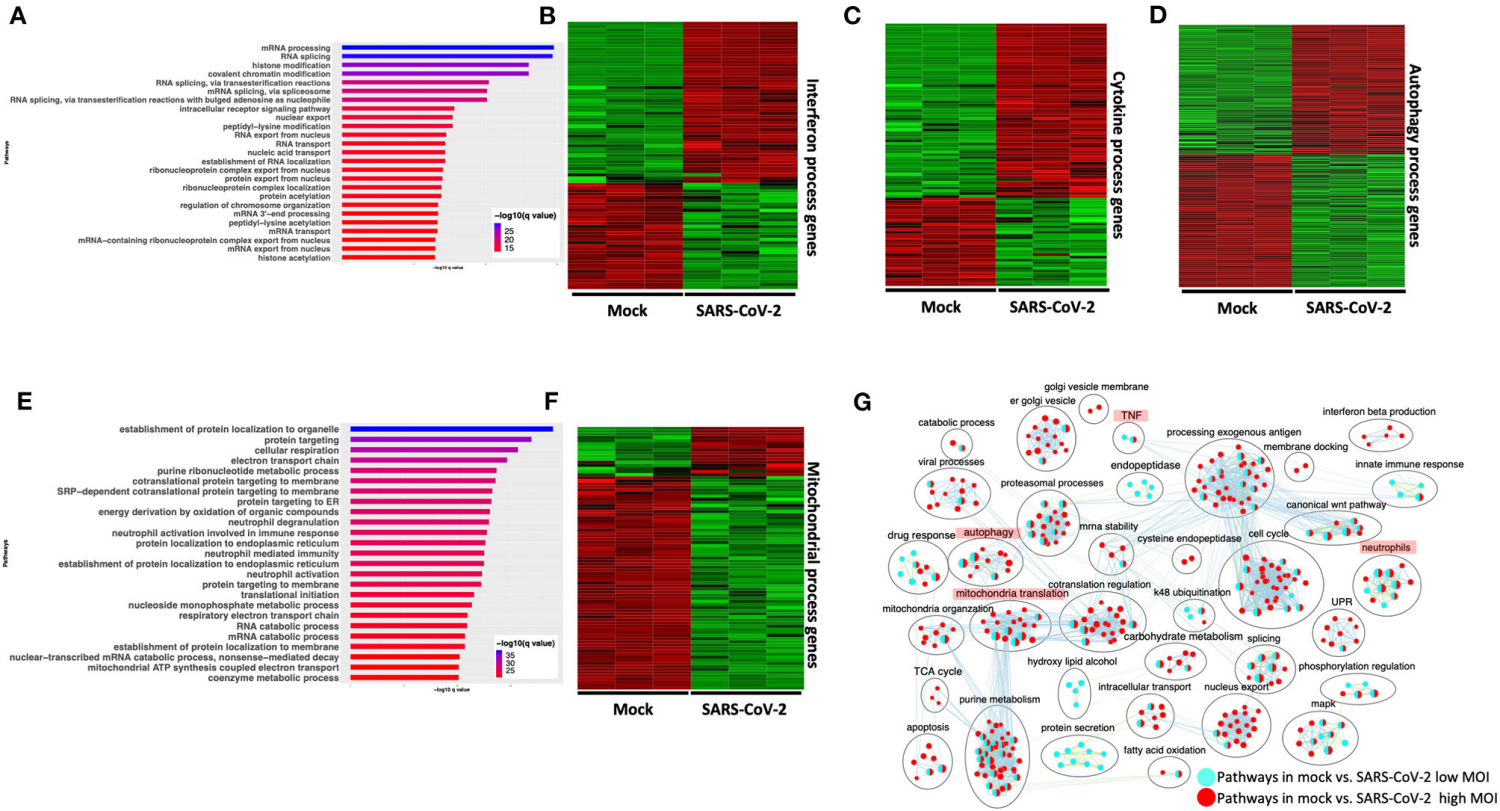
SARS-CoV-2 infection of hACE2 transduced lung epithelial A549 cells impacts expression of genes in interferon, cytokine, and autophagic processes. **(A)** Top 25 pathways from the pathway enrichment analysis of genes upregulated in the SARS-CoV-2-infected A549 cell is presented as a horizontal bar plot, where the *x* axis represents the –log10-transformed *q*-value and the color of the horizontal bar is scaled blue to red representing low to high *q-*values, respectively. **(B)** Heatmap highlighting the expression of genes in the interferon processes in mock and infected cells. The red and green color bands represent up- and downregulated genes, respectively. This heatmap shows that cytokine-related genes were predominantly upregulated in infected cells. **(C)** Heatmap highlighting the expression of genes in the cytokine processes in mock and infected cells. The red and green color bands represent up- and downregulated genes, respectively. This heatmap shows that interferon-related genes were predominantly upregulated in infected cells. **(D)** Heatmap highlighting the expression of genes in the autophagy-related processes in mock and infected cells. The red and green color bands represent up- and downregulated genes, respectively. This heatmap shows that autophagy-related genes were perturbed in infected cells. **(E)** Top 25 pathways from the pathway enrichment analysis of gene upregulated in SARS-CoV-2 infection is presented as a horizontal bar plot, where the *x* axis represents the –log10-transformed *q*-value and the color of the horizontal bar is scaled blue to red representing low to high *q-*values, respectively. **(F)** Heatmap highlighting the expression of genes in the mitochondrial organization and translation in mock and infected cells. The red and green color bands represent up- and downregulated genes, respectively. This heatmap shows that mitochondrial process-related genes were predominantly downregulated in infected cells. **(G)** Pathway enrichment summary map for mock vs. SARS-CoV-2 at high MOI (blue nodes) and low MOI (red nodes) comparisons. Each node represents a pathway/biological process (BP). The node size is proportional to the number of DE genes overlapping with the BP. The nodes that share genes are connected with edges. Single color nodes are pathways that are distinctly enriched by DE genes from one comparison. Two colored nodes are pathways enriched by DE genes from both comparisons. The label above each black circle summarizes the gene ontology (GO) terms of similar BPs present inside the circle. Notable groups of BPs associated with antigen processing, autophagy and mitochondria that were predominantly enriched by DE genes from Mock vs. SARS-CoV-2 (high MOI) comparison and are highlighted in red. BP associated interferon and UPR processes were also predominantly enriched by DE genes from Mock vs. SARS-CoV-2 (high MOI) comparison. MOI, multiplicity of infection; DE, differentially expressed; UPR, unfolded protein response.

Differential gene expression analysis of hACE2 receptor-transduced A549 lung epithelial cell line that was either mock-infected or infected with SARS-CoV-2 at higher viral titer (2MOI) [see Methods (Blanco-Melo et al., 2020)] identified ~8,000 DE genes. The volcano plot profiles both upregulated and downregulated genes in the SARS-CoV-2-infected cells (Supplementary Figure 1A, Supplementary Table 3: Sheet 1). Pathway enrichment analysis of the DE genes showed enrichment in a wide range of BPs (Supplementary Figure 1B, Supplementary Table 4: Sheet 1). These DE genes were classified into upregulated or downregulated following SARS-CoV-2 infection and analyzed by pathway enrichment analysis. Upregulated DE genes annotated to a wide range of pathways, notably including the interferon signaling, NFkB/cytokine signaling processes, and proteasomal degradation (Figure 2A, Supplementary Table 4: Sheet 2). Heatmaps highlight the upregulation of genes in interferon and cytokine processes, and perturbation of genes in the autophagy pathways (Figures 2B–D, respectively). DE genes downregulated in the SARS-CoV-2-infected cells annotated to pathways primarily involving cell cycle and mitochondrial processes (Figure 2E, Supplementary Table 4: Sheet 3). A heatmap shows that the expression of the genes in mitochondria-related processes, electron transport chain, and respiration was mostly downregulated in SARS-CoV-2-infected cells (Figure 2F).

#### SARS-CoV-2: Low Viral Titer vs. High Viral Titer

Differential gene expression analysis of hACE2-transduced A549 cells infected with mock and a 10-fold lower viral titer of SARS-CoV-2 [see Methods (Blanco-Melo et al., 2020)] was also performed. The resulting DE genes could be compared to the DE genes from mock vs. SARS-CoV-2 infection at higher viral titer (Figure 2). Given the exposure of cells to a lower viral titer, the number of DE genes from this comparison was smaller (4,494 genes) vs. the comparison of high titer SARS-CoV-2 against mock (~8,000 genes) (Supplementary Figure 1C, Supplementary Table 3: Sheet 2). Analysis of the 4,494 DE genes showed significant enrichment in inflammation, autophagy and mitochondrial processes (Supplementary Figure 1D, Supplementary Table 4: Sheet 4). To further assess the extent of overlap BPs between low MOI and high MOI A549 cell datasets, the pathway enrichment results were graphically summarized and presented in a single map. This pathway summary map overlays the pathway enrichment results of mock vs. high titer SARS-CoV-2-infected cells on top of the mock vs. low virus titer infected cells to show processes exclusively (single-color nodes) or commonly (double-color nodes) enriched between the two datasets. This analysis confirmed that perturbation in autophagy, inflammation, and mitochondrial processes were enriched by DE genes from both datasets (i.e., high and low MOI infected A549 cells) (Figure 2G).

#### Correlation of Expression Profiles Between Cell Lines and Lung Cell Types

The results presented in the section above and in subsequent sections used gene expression profiles of A549 (that were transduced with hACE2) and Calu3 lung cell lines. How close these cell lines are to lung cells was assessed by evaluating the correlation of the gene expression profiles of cell lines with lung cells using the scRNA-seq information from lung. First, using the scRNA-seq data, cell-type expression profile was computed as the mean expression across cells within each cell type. The top 1,000 genes with the highest variance among the 57 cell-type expression profiles were selected as highly variable genes, which were presumably informative for differentiating the 57 cell types. Next, the expression profiles of lung cell lines were compared with the expression profile of (hACE2-transduced) A549 and Calu3 cell lines. Correlation between the highly variable genes from lung scRNA-seq data and either A549 or Calu3 cells was calculated and plotted (Supplementary Figure 1E). This analysis revealed that the hACE2-transduced A549 cell gene expression strongly correlated with the basal and ionocyte lung cell subpopulations, which both represent lung epithelial cell lineage (Morrisey, 2018; Schiller et al., 2019). These data suggest that the A549 cells' response to SARS-CoV-2 infection likely reflects lung cells' response to the same infection. The Calu3 cells showed a similar pattern but a lower correlation with the lung cell types analyzed (Supplementary Figure 1E).

The correlation analysis (presented above) suggests that the BPs impacted in SARS-CoV-2-infected A549 cells is likely impacted in the SARS-CoV-2-infected lung epithelial cells too. However, given the limitations of analyzing lung cell line data, gene expression analysis of lung samples from patients with severe or mild COVID-19 will help test if these processes are differently impacted depending on the severity of the disease. Together, these results support that SARS-CoV-2 infection impacts the expression of genes involved in the cytokine signaling, autophagy, and mitochondria/respiration.

#### Comparing SARS-CoV-2 Infection in hACE2-Transduced A549 and Calu3 Cell Lines

A number of studies have been published that focused on identifying receptors used by SARS-CoV-2 to delineate viral entry mechanisms. Several of these studies have identified Angiotensin-Converting Enzyme 2 (ACE2) as the receptor that interacts with the SARS-CoV-2 spike protein to mediate viral entry (Li et al., 2007; Shang et al., 2020). Furthermore, TMPRSS2 and TMPRSS4, which are two membrane-bound serine proteases, were found to facilitate viral entry into the cells (Iwata-Yoshikawa et al., 2019; Hoffmann et al., 2020; Zang et al., 2020). Analysis of the gene expression datasets of A549 and Calu3 cells revealed that ACE2 and TMPRSS2 genes are highly expressed in the latter and showed robust gene expression changes in response to SARS-CoV-2 infection (Supplementary Table 3: Sheet 3). To facilitate SARS-CoV-2 infection, A549 cells were transduced with hACE2 vector (Blanco-Melo et al., 2020). The gene expression profile of SARS-CoV-2-infected A549 and Calu3 cells was compared and the correlation of gene expression between infected A549 and Calu3 cells was determined. We found significant correlation (*R* = 0.68, *p* < 2.2e−16) between gene expression of SARS-CoV-2-infected A549 and Calu3 cells (Supplementary Figure 2A). Furthermore, 65% of DE genes from mock vs. SARS-CoV-2 in Calu3 comparison overlapped with DE genes from the respective A549 comparison (Supplementary Figure 2B). Finally, we plotted a pathway enrichment summary map by using the pathway enrichment results from mock vs. SARS-CoV-2 comparison in A549 and Calu3 cells (Supplementary Figure 2C). Overlaying the pathway analysis results from A549 over Calu3 revealed an overlap of a wide range of BPs including the interferon, neutrophils, mitochondrial, and autophagy processes between the two datasets (Supplementary Figure 2C). Together, these results show that, upon ACE2 expression, the gene expression changes in infected A549 cells is highly correlated with infected Calu3 cells.

### Network Analysis Identified Protein–Protein Interaction Subnetworks of Genes Involved in Interferon, Inflammation, and Mitochondrial Translation

#### SARS-CoV-2 vs. Mock: Network Analysis

**Figure 3:**
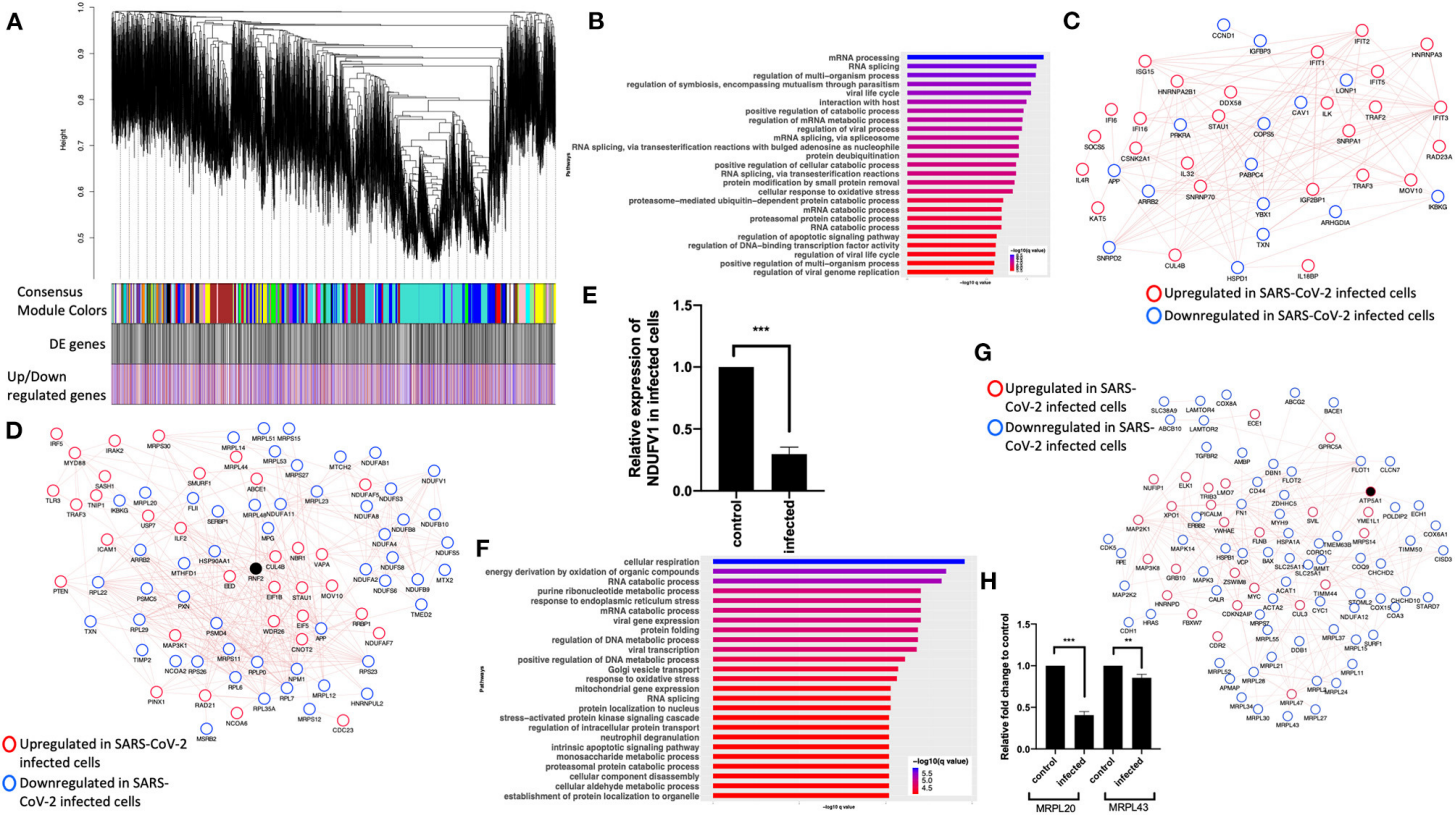
Consensus network analysis of hACE2-transduced mock and SARS-CoV-2-infected (high and low MOI) A549 cells. **(A)** Cluster dendrogram showing correlated genes grouped into clusters marked by different colors on the horizontal block labeled “Consensus Module Colors.” The DE genes in each cluster is marked as a black vertical line in the horizontal block labeled “DE genes.” The up- and downregulated genes are shown as red and blue vertical lines in a block labeled “Upregulated/downregulated genes,” respectively. **(B)** Top pathways from the pathway enrichment analysis of correlated DE genes in the blue module is presented as a horizontal bar plot, where the *x* axis represents the –log10-transformed *q*-value and the color of the horizontal bar is scaled blue to red representing low to high *q*-values, respectively. **(C)** Protein–protein interaction (PPI) subnetworks in the blue module is presented where each node represents a gene and the border color of the nodes indicate upregulation (red) and downregulation (blue) in SARS-CoV-2-infected A549 cells (high MOI) compared to mock-infected cells. The edge between the nodes indicate interaction based on the GeneMANIA database information. The network shows a highly connected interactome of interferon-stimulated genes (ISGs) that are coordinately upregulated in infected cells. **(D)** Another PPI subnetwork identified in the blue module shows several highly interconnected mitochondrial ribosomal (MRP) and complex I (NDUF) genes. Each node represents a gene and the border color of the nodes indicate upregulation (red color) and downregulation (blue) in SARS-CoV-2-infected A549 cells (high MOI) compared to mock-infected cells. Additional interactome of MYD88 TRAF3 and TLR3 genes that were coordinately upregulated in infected cells can be seen (top left). **(E)** NDUFV1 transcript level reported as fold change in beta-coronavirus-infected HCT-8 cells compared to control (no infection) HCT-8 cells. Student's *t*-test *p*-value = 2E-07. **(F)** Top pathways from the pathway enrichment analysis of correlated DE genes in the turquoise module is presented as a horizontal bar plot, where the *x* axis represents the –log10-transformed *q*-value and the color of the horizontal bar is scaled blue to red representing low to high *q-*values, respectively. **(G)** MRPL20 and MRPL43 transcript levels reported as fold change in beta-coronavirus-infected HCT-8 cells compared to control (no infection) HCT-8 cells. Student's *t*-test *p*-value = 8E-08 and 0.007. **(H)** A PPI subnetwork of correlated DE genes in the turquoise module shows a well-connected interactome of genes encoding mitochondrial ribosomal proteins, mitochondrial coiled-coil-helix-coiled-coil-helix domain proteins, and cytochrome oxidase. Each node represents a gene and the border color of the nodes indicates upregulation (red color) and downregulation (blue) in SARS-CoV-2-infected cells (high MOI) compared to mock-infected cells. DE, differentially expressed; MOI, multiplicity of infection.

To further understand the potential BPs in play during SARS-CoV-2 infection, we performed a consensus weighted gene coexpression network analysis (WGCNA) (Langfelder and Horvath, 2008) on combined, batch-corrected (see Methods), gene expression values of mock and SARS-CoV-2 infected at low and high titer cells, to identify clusters/modules of correlated gene. WGCNA identified more than 47 coexpression modules. The overlap of genes in each of these modules with significant DE genes from mock vs. SARS-CoV-2 infected at high titer is presented in the cluster dendrogram where each correlated module is represented by a color, and their overlap with DE genes is shown in horizontal bars (Figure 3A, Supplementary Table 1).

First, pathway enrichment analysis of the correlated DE genes in the blue module showed significant annotation to the mitochondria, immunity, and mRNA/transcription processes (Figure 3B, Supplementary Table 4: Sheet 5). Using the GeneMANIA (Franz et al., 2018) database, protein–protein interaction (PPI) subnetworks for the DE genes in this module/cluster were identified. This analysis identified two PPI subnetworks of genes involved in interferon signaling (Figure 3C), TLR3 and MYD88 (Figure 3D), and mitochondrial translation and complex I genes (Figure 3D). Consistent with the expression level of complex I genes in the RNA-seq data, the mRNA level of NDUFV1 was down in beta-coronavirus-infected HCT-8 cells when measured by quantitative real-time polymerase chain reaction (qRT-PCR) (Figure 3E). Together, from these data, we concluded that the interferon signaling and inflammation genes were upregulated, and mitochondrial genes were downregulated in SARS-CoV-2-infected cells.

Next, pathway enrichment analysis of DE genes from the turquoise module revealed significant annotation to viral gene expression and apoptosis processes (Figure 3F, Supplementary Table 4: Sheet 6). Using GeneMANIA database, a PPI subnetwork of genes encoding the mitochondrial ribosomal proteins that were mostly downregulated in SARS-CoV-2-infected cells was also identified (Figure 3G). Consistent with the RNA-seq data, mRNA levels of the mitochondrial ribosomal genes MRPL20 and MRPL43 were downregulated in beta-coronavirus-infected HCT-8 cells when measured by qRT-PCR (Figure 3H). Together, these data suggest that SARS-CoV-2 infection results in a coordinated change in the interferon signaling, inflammation, and mitochondrial processes.

### Gene Expression Changes Associated With SARS-CoV-2 Infection Is Distinct From IAV Infection With Minor Overlaps

#### SARS-CoV-2 vs. IAV

**Figure 4:**
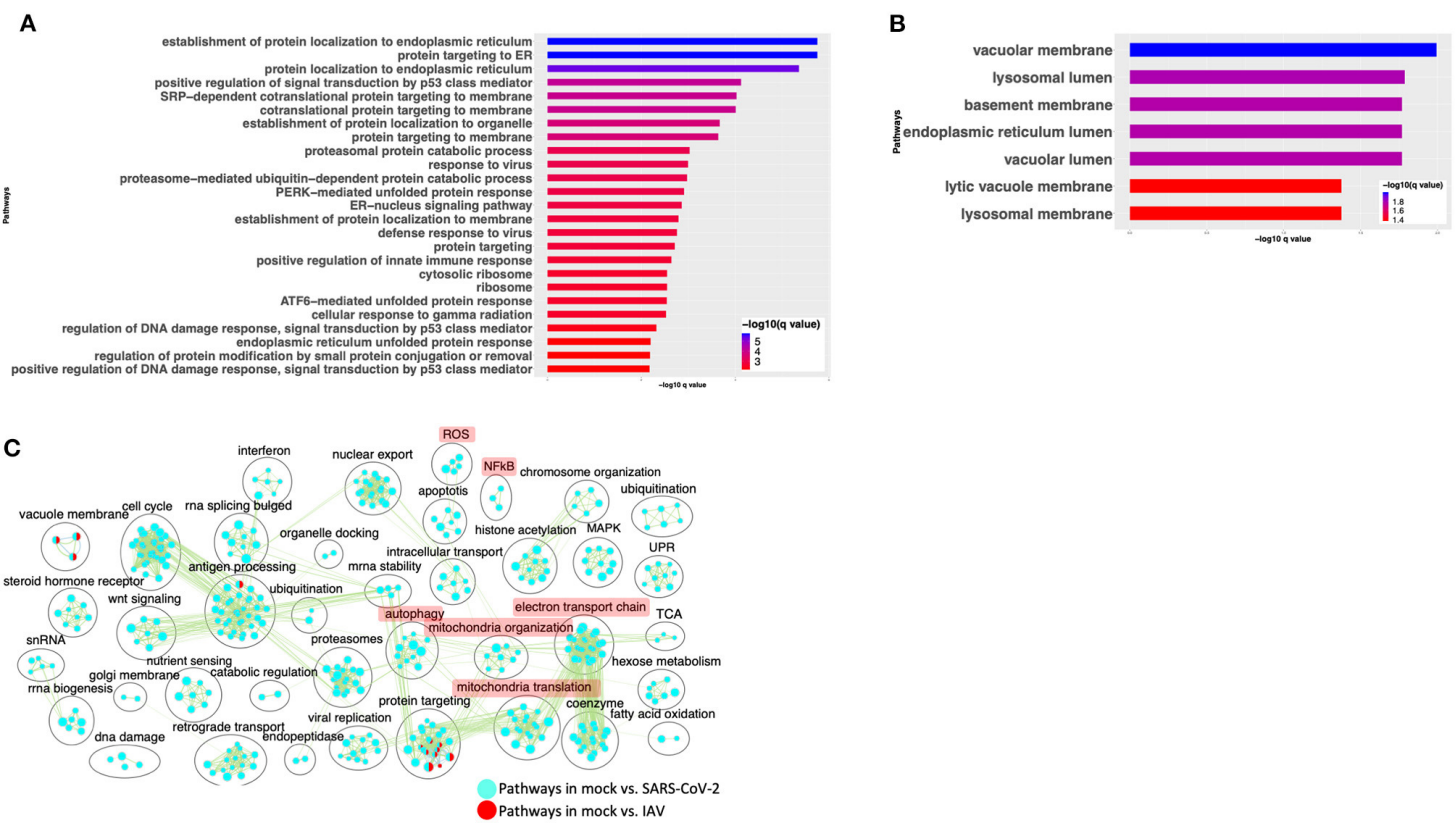
SARS-CoV-2 infection of A549 lung epithelial cells results in distinct gene expression changes that are not seen in IAV infection. **(A)** Top 25 pathways from the pathway enrichment analysis of DE genes upregulated in IAV-infected cells is presented as a horizontal bar plot, where the *x* axis represents the –log10-transformed *q-*value and the color of the horizontal bar is scaled blue to red representing low to high *q*-values, respectively. **(B)** Top 25 pathways from the pathway enrichment analysis of DE genes downregulated in IAV-infected cells is presented as a horizontal bar plot, where the *x* axis represents the –log10-transformed *q-*value and the color of the horizontal bar is scaled blue to red representing low to high *q-*values, respectively. **(C)** Pathway enrichment summary map for mock vs. SARS-CoV-2 (blue nodes) and mock vs. IAV (red nodes) comparisons. Each node represents a pathway/biological process (BP). The node size is proportional to the number of DE genes overlapping with the BP. The nodes that share genes are connected with edges. The label above each black circle summarizes the gene ontology (GO) terms of similar BPs present inside the circle. Single-color nodes are pathways that are distinctly enriched by DE genes from one comparison. Two-colored nodes are pathways enriched by DE genes from both comparisons. Notable groups of BPs associated with NFkB, ROS, autophagy and mitochondria are highlighted in red. DE genes from Mock vs. SARS-CoV-2 comparison exclusively enriched in highlighted BPs.

To compare the expression profile of SARS-CoV-2-infected cells with other virus-infected cells, DE analysis of mock- vs. IAV-infected cells was performed and the up- and downregulated genes are presented in a volcano plot (Supplementary Figure 3A, Supplementary Table 3: Sheet 4). The pathway analysis of the DE genes from this comparison showed enrichment in protein translation, localization, and anti-viral responses (Supplementary Figure 3B, Supplementary Table 4: Sheet 7). Additionally, genes upregulated in the IAV-infected cells annotated to pathways for virus response, protein trafficking, and unfolded protein response (UPR) (Figure 4A, Supplementary Table 4: Sheet 8). Genes that were downregulated in IAV-infected cells enriched in vacuole- and lysosome-related processes (Figure 4B, Supplementary Table 4: Sheet 9). Interestingly, few DE genes from the mock vs. SARS-CoV-2 overlapped with the DE genes from mock vs. IAV comparison (Supplementary Figure 3C).

A pathway enrichment summary map was created by overlaying the pathway enrichment results of the mock vs. IAV comparison on top of the mock vs. SARS-CoV-2 comparison. Consistent with the DE genes comparison (Supplementary Figure 3C), the enrichment map also highlighted little overlap of pathways between the two comparisons. DE genes from both comparisons commonly enriched in a subset of pathways associated with protein trafficking (Figure 4C). Furthermore, only a subset of the interferon pathway genes and a few chemokine genes that were upregulated in SARS-CoV-2-infected cells were also upregulated in IAV-infected cells, while the autophagy and inflammation genes remained mostly unchanged in the latter. Therefore, upregulation of cytokine/inflammation, changes in autophagy, and downregulation of the mitochondrial processes were uniquely observed in SARS-CoV-2-infected cells. Upregulation of DE genes involved in the cytokine/inflammation processes is consistent with cytokine storm observed in severe cases of COVID-19 patients. Since these observations were made by analyzing the gene expression changes in a lung cell line, future studies profiling gene expression changes in severe COVID-19 patients' lung samples will be needed to confirm these findings.

### SARS-CoV-2-Infected Cells Share Some Gene Expression Signature With MERS-CoV-Infected Cells With Few Exceptions

#### SARS-CoV-2 vs. MERS-CoV

**Figure 5:**
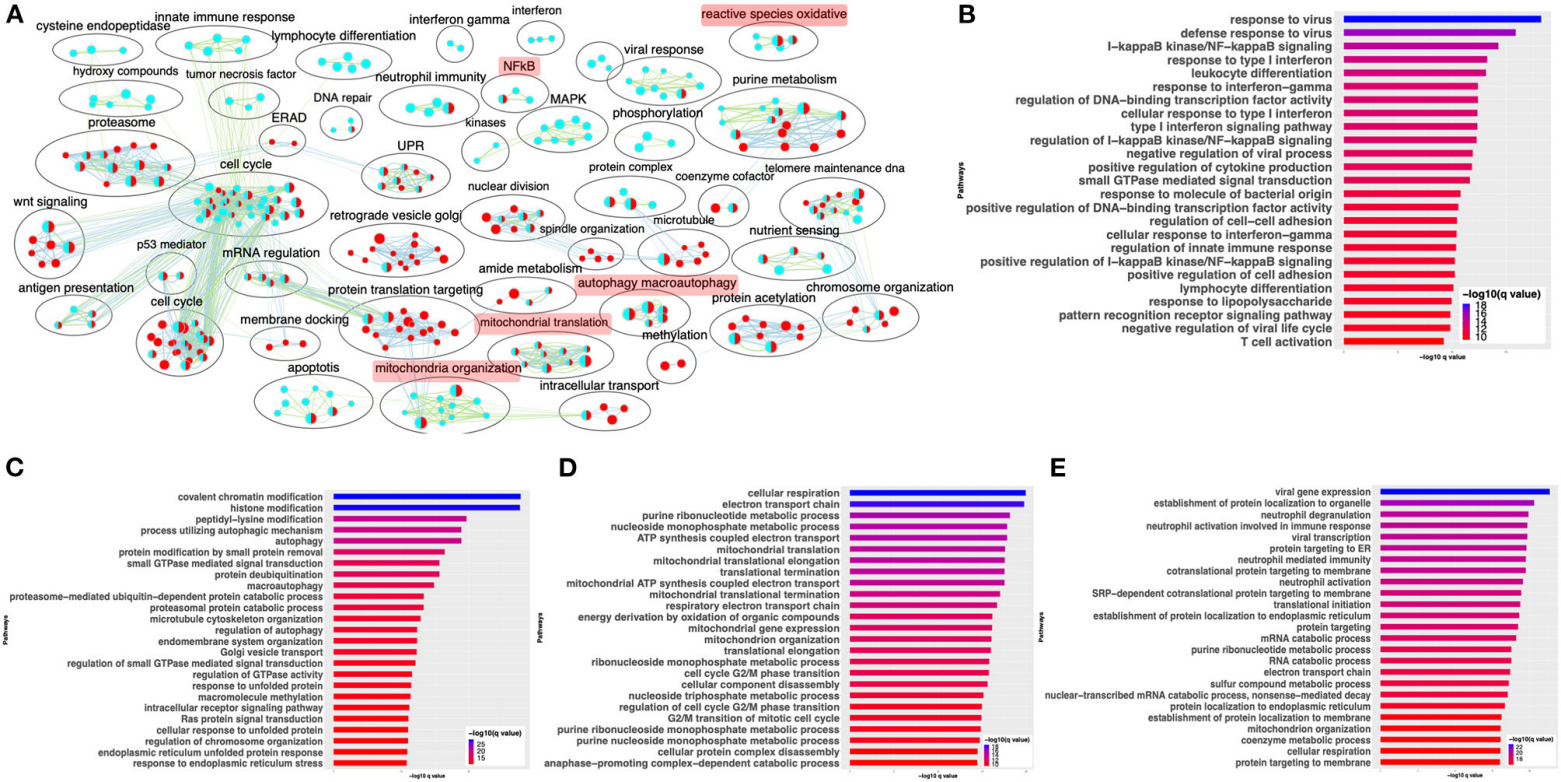
SARS-CoV-2 and MERS-CoV infection have some common and some distinct gene expression signatures. **(A)** Pathway enrichment summary map for mock vs. SARS-CoV-2 (blue nodes) and mock vs. MERS-CoV (red nodes) comparisons in Calu 3 cells. Each node represents a pathway/biological process (BP). The node size is proportional to the number of DE genes overlapping with the BP. The nodes that share genes are connected with edges. The label above each black circle summarizes the gene ontology (GO) terms of similar BPs present inside the circle. Single-color nodes are pathways that are distinctly enriched by DE genes from one comparison. Two-colored nodes are pathways enriched by DE genes from both comparisons. Notable groups of BPs associated with immunity, ROS, autophagy, and mitochondria are highlighted in red. These notable groups of BPs were commonly enriched by DE genes from both SARS-CoV-2 and MERS-CoV comparisons. The DE genes from mock vs. SARS-CoV-2 comparison predominantly enriched in inflammation and immunity-related processes. **(B)** Top 25 pathways from the pathway enrichment analysis of DE genes upregulated in SARS-CoV-2-infected Calu3 cells is presented as a horizontal bar plot, where the *x* axis represents the –log10-transformed *q-*value and the color of the horizontal bar is scaled blue to red representing low to high *q-*values, respectively. **(C)** Top 25 pathways from the pathway enrichment analysis of DE genes upregulated in MERS-CoV-infected Calu3 cells is presented as a horizontal bar plot, where the *x* axis represents the –log10-transformed *q-*value and the color of the horizontal bar is scaled blue to red representing low to high *q-*values, respectively. **(D)** Top 25 pathways from the pathway enrichment analysis of DE genes downregulated in SARS-CoV-2-infected Calu3 cells are presented as a horizontal bar plot, where the *x* axis represents the –log10-transformed *q-*value and the color of the horizontal bar is scaled blue to red representing low to high *q-*values, respectively. **(E)** Top 25 pathways from the pathway enrichment analysis of DE genes downregulated in MERS-CoV-infected Calu3 cells is presented as a horizontal bar plot, where the *x* axis represents the –log10-transformed *q-*value and the color of the horizontal bar is scaled blue to red representing low to high *q-*values, respectively. DE: differentially expressed.

Comparison of gene expression profiles revealed that SARS-CoV-2-infected cells are distinct from those of IAV-infected cells (Figure 4). Although these are both viruses, they are not phylogenetically close. Therefore, we next compared the gene expression profiles of SARS-CoV-2- and MERS-CoV-infected cells, since both are hCoVs. DE analysis of the mock- vs. SARS-CoV-2-infected Calu3 lung carcinoma cells identified several up- and downregulated genes (Supplementary Figure 4A, Supplementary Table 3: Sheet 3). Pathway enrichment analysis showed annotation of the DE genes to cell cycle, inflammation, and apoptosis processes (Supplementary Figure 4B, Supplementary Table 4: Sheet 10). A pathway enrichment summary map for mock vs. SARS-CoV-2 and mock vs. MERS-CoV comparisons was generated to assess the extent of overlap of pathways between the two datasets (Figure 5A). Notably, the DE genes from both comparisons enriched in the mitochondria, autophagy, cell cycle, and UPR processes. However, DE genes from mock vs. SARS-CoV-2 comparison predominantly enriched in inflammation, cytokine signaling, and immunity-related processes (Figure 5A). Consistently, genes upregulated in the SARS-CoV-2-infected Calu3 cells enriched in inflammation and nuclear factor kappaB (NFkB) processes (Figure 5B, Supplementary Table 4: Sheet 11), while upregulated genes from both hCoV-infected cells annotated to protein trafficking and small GTPase signaling (Figures 5B,C, Supplementary Table 4: Sheets 11 and 12). On the other hand, genes downregulated in both comparisons commonly annotated to mitochondrial processes (Figures 5D,E, Supplementary Table 4: Sheets 13 and 14). These findings suggest that perturbation of autophagy and mitochondrial genes are common gene expression signatures associated with hCoV infection, but the SARS-CoV-2 virus almost exclusively impacts the cytokine/inflammatory processes in the lung cells. It is likely that perturbation of mitochondrial processes and autophagy may lead to a dysfunctional immune response (Won et al., 2015; Jang et al., 2019). Further studies will be required to understand if and how these processes may contribute to inflammation during SARS-CoV-2 infection.

### Validation of Findings From Cell Line Data in SARS-CoV-2-Positive Human Samples

#### Comparing SARS-CoV-2-Positive vs. -Negative Human Nasopharyngeal Expression Profile With A549 Dataset

**Figure 6:**
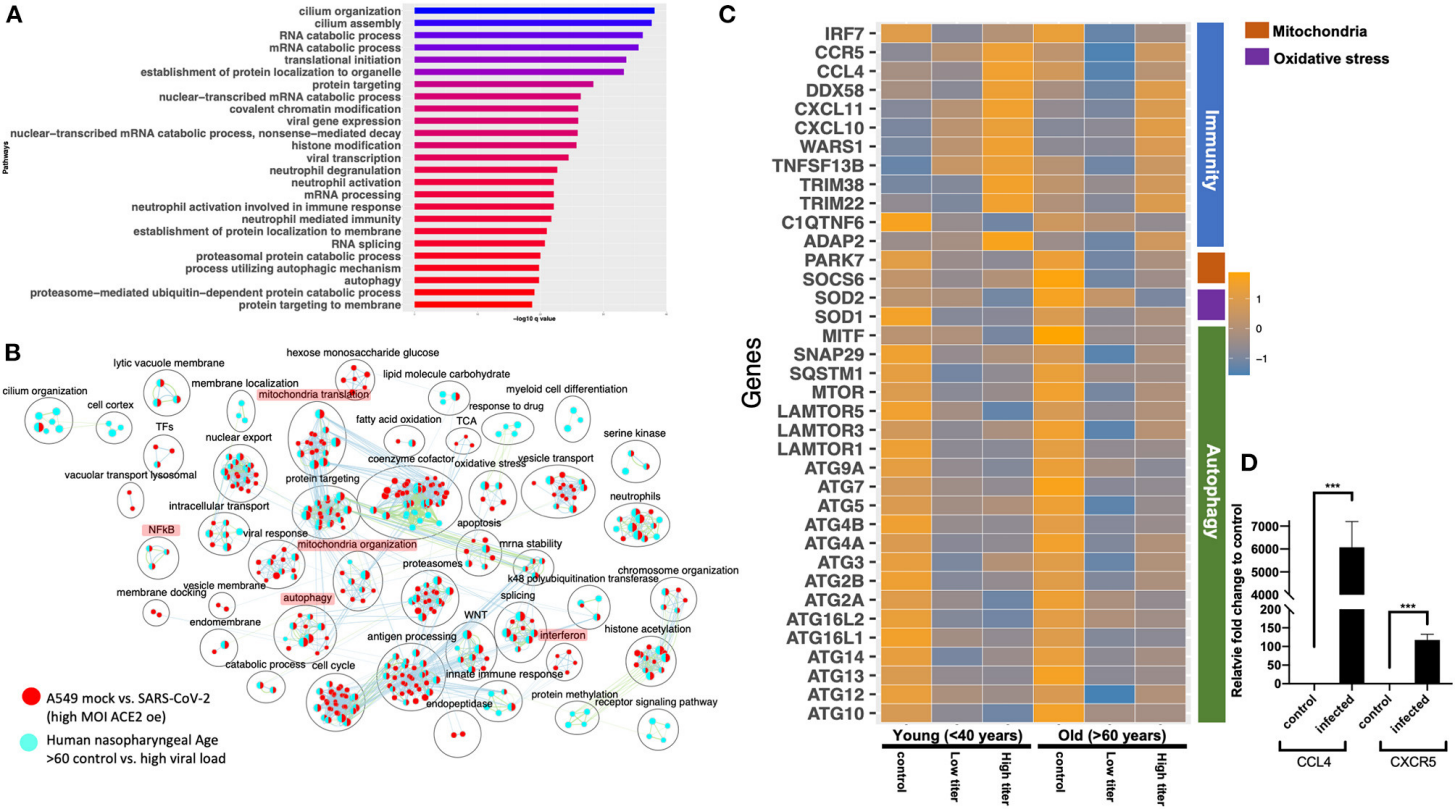
Gene expression profiling of human nasopharyngeal samples. **(A)** Top 25 pathways from the pathway enrichment analysis of DE genes from control vs. high viral load positive old age human samples is presented as a horizontal bar plot, where the *x* axis represents the –log10-transformed *q-*value and the color of the horizontal bar is scaled blue to red representing low to high *q-*values, respectively. **(B)** Pathway enrichment summary map for control vs. high viral load positive human samples (blue nodes) and mock vs. SASR-CoV-2 (red nodes) comparisons in hACE2-transduced A549 cells. Each node represents a pathway/biological process (BP). The node size is proportional to the number of DE genes overlapping with the BP. The nodes that share genes are connected with edges. The label above each black circle summarizes the gene ontology (GO) terms of similar BPs present inside the circle. Single-color nodes are pathways that are distinctly enriched by DE genes from one comparison. Two-colored nodes are pathways enriched by DE genes from both comparisons. Notable groups of BPs associated with immunity, autophagy, and mitochondria are highlighted in red. These notable groups of BPs were commonly enriched by DE genes from both comparisons. **(C)** Heatmap of the mean expression values of the indicated genes in young and old human samples that were negative (control) or positive with either high or low viral loads of SARS-CoV-2 virus. The indicated genes are broadly grouped into four different processes using a vertical bar present on the right side of the heatmap. The orange and blue color bands represent upregulated and downregulated genes, respectively. **(D)** CCL4 and CXCR5 chemokine and receptor transcript levels reported as fold change in beta-coronavirus-infected HCT-8 cells compared to control (no infection) HCT-8 cells. Student's *t*-test *p*-value = 3E-04 and 1.7E-05.

The gene expression profiling of the A549 and Calu3 cell line data revealed that perturbations in inflammatory, autophagy, and mitochondrial processes were unique to coronavirus infections. To validate the findings from SARS-CoV-2-infected lung cell lines, we analyzed the gene expression profiles of SARS-CoV-2-positive human nasopharyngeal samples. DE analysis of SARS-CoV-2-positive vs. -negative samples revealed up- and downregulated genes (Supplementary Table 3: Sheet 5). Concurrent with the cell line data, the pathway enrichment analysis of the DE genes revealed significant annotation to inflammation, autophagy, and mitochondrial processes (Supplementary Figure 5A, Supplementary Table 4: Sheet 15). Approximately 60% of the DE genes from the SARS-CoV-2-infected human samples comparison overlapped with the infected A549 cell data (Supplementary Figure 5B). Of this, ~50% of the DE genes were concurrently downregulated in both datasets (i.e., human samples and A549 cells) and significantly annotated to the autophagy, immunity, and mitochondrial processes (Supplementary Figure 5C). Since age (Wang D. et al., 2020) and viral load (Liu et al., 2020) can determine the severity of the COVID-19 outcome, the human samples were further classified into young (<40 years) and old (>60 years) with low or high viral load positive samples. Consistent with the SAR-CoV-2-positive vs. -negative human data, the DE genes from the control vs. high viral load in both old and young subjects significantly annotated to inflammation, autophagy, and mitochondrial processes [Figure 6A (old subject), Supplementary Table 3: Sheets 7 and Sheet 9, and Supplementary Table 4: Sheets 16 and 17]. When the pathway enrichment result from the old human subjects (that were high viral load positive) was overlaid on the pathway enrichment results from A549 cells, transduced hACE2 infected with high MOI of SARS-CoV-2, autophagy, NFkB, oxidative stress, and mitochondrial processes were commonly perturbed in both datasets (Figure 6B). Together, these observations suggest that across cell types, SARS-CoV-2 infection alters the cells' autophagy and mitochondrial processes and that perturbations in these processes may impede an effective immune response leading to severe outcomes.

#### Delineating the Age and Viral Load Impact on SARS-CoV-2 Response in Human Nasopharyngeal Samples

The human nasopharyngeal samples were classified into young and old samples with low or high viral load to delineate either age specific or viral load specific gene expression changes in response to infection. The analysis in control vs. infected (with either low or high viral load) young or old samples helped identify DE genes that significantly annotated to autophagy, neutrophils, and mitochondrial processes (Supplementary Table 3: Sheets 6–9), which is consistent with the pathway enrichment results from SARS-CoV-2-infected A549 cell line data (Figure 2). However, in-depth gene expression analysis of the human data revealed some concurrent gene expression changes between cell line and human datasets, and some sample specific changes. Genes encoding the mTOR (Figure 6C), mitochondrial ribosomal genes, mitochondrial complex I genes, and lysosome acidification genes were downregulated in all of the SARS-CoV-2-positive human samples as well as in SARS-CoV-2-infected A549 cells (Supplementary Table 3: Sheet 1). Additionally, autophagy initiation, nucleation genes, p62 (SQSTM1), SNAP29, and MITF were specifically downregulated in SARS-CoV-2-positive human samples, which indicates decreased autophagic flux in infected samples (Figure 6C and Supplementary Figure 6D). Furthermore, antioxidant encoding SOD1 (Fukai and Ushio-Fukai, 2011) gene, the oxidative stress sensor PARK7 (DJ-1) (Wang et al., 2016), and mitochondria fission promoting SOCS6 (Lin et al., 2013) were also downregulated in infected human samples (Figure 6C). Finally, several cytokine/inflammation process genes were downregulated in infected human samples, which was opposite to the upregulation of these genes in SARS-CoV-2-infected A549 cells (Supplementary Table 3: Sheet 1).

There were several age- and viral load-dependent gene expression changes observed in the human nasopharyngeal samples, which are described below. Several interferon pathway genes were upregulated in high viral infected young and old samples. However, the upregulation of these genes in high viral load positive old samples was muted compared to the corresponding young samples (Supplementary Figure 6A). The difference in interferon genes induction was most prominent in low viral load positive samples, as unlike the young samples where most of these genes were upregulated, in old samples, these interferon genes were significantly downregulated (Supplementary Figure 6A). Similarly, ADAP2 [which is an interferon-stimulated gene (ISG)], antiviral tripartite motif family E3 ligase TRIM38, chemokine CCL4 and its receptor CCR5, and WARS1 encoding the Tryptophanyl-tRNA Synthetase were all robustly down in old, low viral dose positive samples compared to the age-matched control (Figure 6C). In response to the high viral titer infection, both CCL4 and CXCR5 were upregulated more robustly in young subjects compared to old subjects. Consistent with the expression in the nasopharyngeal samples, robust upregulation of CCL4 and CXCR5 mRNAs was also observed in HCT-8 cells infected with the beta-coronavirus compared to the control cells by qRT-PCR (Figure 6D). Upregulation of chemokine signaling may be a common host cell response to coronaviruses. Together, these data suggest age-specific differences in host transcriptional response to SARS-CoV-2 infection. However, further analysis of the infected lung samples from old and young patients will be required to test if these differences may be causing more severe outcomes in older patients.

### Gene Expression Analysis of a Severe COVID-19 Lung Sample Shows Exaggerated Immune/Inflammation Response

#### Healthy vs. COVID-19 Lung Biopsy Samples

The gene expression analysis of the SAR-CoV-2-infected A549 and Calu3 cell lines revealed upregulation of the cytokine/inflammatory processes. However, this observation was inconsistent with the human nasopharyngeal expression profile where inflammatory process genes were downregulated. This difference could arise due to disparate cell type being compared, or that severe inflammatory symptoms may arise in severe case of COVID-19. Since the human nasopharyngeal data did not include clinical symptoms indicating the severity of COVID-19 in the samples whose sequence were analyzed, we could not stratify the samples based on disease severity. However, to test if the cytokine/inflammatory processes were also impacted in COVID-19 lungs, RNA-seq data from the healthy and COVID-19 lung biopsy were analyzed. It should be noted that COVID-19 lung biopsies were technical replicates and therefore statistical significance of this analysis is limiting. Future studies involving bigger sample size will be required to confirm these observations. Nevertheless, the gene expression profile of a SARS-CoV-2-infected lung was distinct from healthy lungs with up- and downregulated genes highlighted in the volcano plot (Supplementary Figure 7A, Supplementary Table 3: Sheet 10). The pathway enrichment summary map and the plot showed that the DE genes predominantly annotated to the inflammation, ROS, and leukocyte/monocyte-related pathways (Supplementary Figure 7B). Furthermore, the DE genes upregulated in the COVID-19 lungs enriched in the anti-viral response processes, cytokine secretion, immune cell proliferation/migration, and inflammation (Supplementary Figure 7C, Supplementary Table 4: Sheet 18). The downregulated genes were significantly enriched in protein trafficking, RNA metabolism, and oxygen-sensing processes (Supplementary Figure 7D, Supplementary Table 4: Sheet 19). It is likely that perturbations in the oxygen-sensing processes are reflective of the severe respiratory distress often seen in severe COVID-19 patients due to reduced oxygenation ability of the failing lungs.

## Discussion

Highly pathogenic hCoVs are known to infect the lower respiratory airways and cause severe acute respiratory syndrome (SARS) (Channappanavar and Perlman, 2017). The recently discovered SARS-CoV-2 virus is the cause of COVID-19 (Tammaro et al., 2020). The clinical manifestations of this virus infection include fever, cough, fatigue, respiratory distress, and cardiac injury (Chen N. et al., 2020; Guan et al., 2020; Huang et al., 2020). While some patients with COVID-19 suffered from mild symptoms, other patients had increasingly life-threatening symptoms (Guan et al., 2020). Age and underlying medical conditions such as diabetes and hypertension are likely to determine the severity of the symptoms (Wang D. et al., 2020). Analyzing the gene expression profiles of host cells infected with SARS-CoV-2 will be necessary to decipher the subcellular functions perturbed by this virus and to inform drug development strategies.

Here, we present an in-depth differential expression analysis of A549 and Calu3 cell lines, comparing mock to infection with either SARS-CoV-2, IAV, or MERS-CoV. Since A549 cells lacked expression of ACE2, TMPRSS2, or TMPRSS4 that is required for SARS-CoV-2 viral entry into the cells (Iwata-Yoshikawa et al., 2019; Hoffmann et al., 2020; Zang et al., 2020), A549 cells transduced with hACE2 were used. Upon SARS-CoV-2 infection at low and high MOI, viral transcripts were detected in these cells indicating infection (Blanco-Melo et al., 2020). Concurrently, we also observed strong correlation between SARS-CoV-2-infected Calu3 and hACE2-transduced A549 cells, suggesting that SARS-CoV-2 infection of the lung cell lines results in similar gene expression profile. Furthermore, we report that (i) SARS-CoV-2 infection impacted the expression of genes in inflammation, cell cycle, reactive oxygen species (ROS), autophagy, and mitochondrial processes, which were absent in IAV-infected cells; (ii) while perturbation in autophagy and mitochondrial processes is common in hCoV infections (SARS-CoV-2 and MERS-CoV), we found that increased expression of the inflammatory/cytokine signaling genes was exclusively observed in SARS-CoV-2-infected lung cells; (iii) coexpression network analysis helped identify a cluster of genes involved in inflammation and mitochondrial translation process that were either coordinately up- or downregulated in SARS-CoV-2-infected cells, respectively. Together, these data suggest that perturbation in the autophagy, mitochondrial processes in SARS-CoV-2-infected lung cells could hinder an effective immune response (Won et al., 2015; Jang et al., 2019) and increase inflammation, which is often seen in severe COVID-19 patients suffering from cytokine storm (Channappanavar and Perlman, 2017; Mehta et al., 2020). Since these conclusions were made using the data from virus-infected lung cell lines, the correlation between these cells' expression profiles and marker gene expression from different lung cell types were determined. While the A549 cells showed robust correlation with lung epithelial lineage basal and ionocyte cells, Calu3 cells showed a similar pattern but lower correlation with these cell types. Therefore, the processes delineated in SARS-CoV-2 A549 cells likely represent the lung epithelial cells response to SARS-CoV-2 infection. To further substantiate the cell line findings, the gene expression profiles of SARS-CoV-2-positive human nasopharyngeal samples were used for validation. Analysis of control vs. SARS-CoV-2-positive samples helped identify DE genes that significantly annotated to the autophagy, NFkB, oxidative stress, and mitochondrial processes. Using the patient information available from this dataset, the samples were grouped into young (<40 years) and old (>60 years) that were either positive with low or high viral load. Comparing the gene expression and pathway enrichment results of old age samples with that of A549 high MOI data revealed a wide range of BPs that were commonly perturbed in both datasets. Therefore, this analysis has delineated several BPs, discussed in more detail below, that are impacted in the SARS-CoV-2-infected host cells.

Some complement genes (C1S and C1R) were specifically upregulated in high viral titer SARS-CoV-2-infected cell lines. Consistently, the C1q/TNF-related protein 6, a glycoprotein that regulates complement activation, was downregulated in both SARS-CoV-2-infected cells and human samples. This gene is implicated in arthritis, and intra-articular injection of the recombinant C1qTNF6 protein was shown as an effective strategy in improving arthritis and inflammation in C1qtnf6–/– mice (Murayama et al., 2015). An elevated complement response could likely lead to excessive inflammation, which was also observed in MERS-CoV infection of the hDPP4-transgenic mouse model (Jiang et al., 2018). Additionally, several past studies have highlighted the interplay between the complement and coagulation systems (Skoglund et al., 2010; Oikonomopoulou et al., 2012). It is likely that the increased thrombosis in COVID-19 patients (Bikdeli et al., 2020) is a result of excessive complement activation. Further assessment of complement activation in COVID-19 patients will be required to confirm this. Together, these observations suggest that inhibition of the complement system as potential treatment strategies could be tested.

Infection of A549 cells with SARS-CoV-2 at higher viral titer perturbed autophagy; upregulated genes in the interferon, cytokine, nuclear factor kappaB (NFkB), and reactive oxygen species (ROS) processes; and downregulated genes in the mitochondrial and electron transport chain processes. Consistently, analysis of DE genes in one of the correlated clusters from WGCNA showed significant enrichment in the interferon signaling processes. Additionally, GeneMANIA analysis of the correlated DE genes in two modules revealed PPI subnetworks of genes involved in ISGs and NFkB, which were both mostly upregulated in the infected cells. In addition to the ISGs, the JAK-STAT signal transduction genes, which play critical role in type I cytokine (such as IL6) signaling and inflammation (Leonard and O'shea, 1998; O'brown et al., 2015; Banerjee et al., 2017), were also upregulated in the SARS-CoV-2-infected A549 cells (Figure 4C). IL6, a pleotropic cytokine, was shown to be elevated in critically ill COVID-19 patients (Chen X. et al., 2020). Consistently, IL6 was upregulated in the SARS-CoV-2-infected cells. IL6 acts via the JAK-STAT signaling through SOCS3 protein kinase (also upregulated in SARS-CoV-2-infected cells) to activate the immune response (Brocke-Heidrich et al., 2004). Excessive IL6 causes excessive inflammation as seen in arthritis (Srirangan and Choy, 2010). However, the inflammatory/cytokine gene expression profile in the virus-infected human nasopharyngeal samples was distinct from the cell line data. In the infected nasopharyngeal samples, most of the cytokine/inflammatory process genes were significantly downregulated compared to the control. These discordant inflammatory gene expression profiles between two datasets may be due to different cell types being compared (A549 is a lung cell line, and human nasopharyngeal samples are predominantly squamous epithelial cells), or the upregulation of cytokine/inflammatory processes genes in SARS-CoV-2-infected A549 cells may represent a severe COVID-19 infection state. Due to lack of clinical information describing the disease state of subjects whose nasopharyngeal samples were analyzed, we could not test this possibility in the nasopharyngeal dataset. However, analysis of the COVID-19 lung biopsy samples revealed significant upregulation of genes enriched in cytokine/inflammatory processes. Therefore, upregulation of IL6 and NFkB genes may contribute to the inflammatory symptoms observed in severe COVID-19 patients (Channappanavar and Perlman, 2017; Mehta et al., 2020). These data support a central role for cytokine signaling in COVID-19 pathogenesis. Treatment strategies aimed at mitigating the cytokine effects or complement system could be tested in treatment of COVID-19. One such clinical trial aimed at mitigating the IL6 effects is already underway (NCT04322773). Furthermore, another study showed decreased mortality in patients treated with tocilizumab, which blocks IL6 (Somers et al., 2020).

Analysis of young (<40 years) and old (>60 years) nasopharyngeal samples also revealed some age-specific changes in the gene expression profile. Notably, the ISGs (IFIT1, IFIT2, and IFIT3) were upregulated in both low and high viral load SARS-CoV-2-positive young samples. Upregulation of these genes in high viral load infected old age samples were, however, less robust. Furthermore, in the low viral load positive old samples, most of the interferon genes (except IFIT3) were downregulated. In addition to the interferon genes, ADAP2, which is an ISG (Shu et al., 2015), TRIM5, which is a retroviral restriction factor (De Silva and Wu, 2011), tripartite motif TRIM22, and TRIM38 were significantly downregulated in low viral load positive old samples compared to age-matched control. The latter two genes are involved in innate immunity and in restricting viral infections (Barr et al., 2008; Lian and Sun, 2017). Moreover, the Tryptophanyl-tRNA Synthetase encoding WARS1, which stimulates immunity against viral infection (Lee et al., 2019), chemokines CXCL11, CCL4, and CCL4 receptor CCR5 were also significantly downregulated in low viral load positive old samples compared to age-matched control. Chemokine CXCL10 expression was unchanged in the low viral load positive old samples. All these chemokines and the receptor were upregulated in the low viral load positive young samples. Consistent with this, qRT-PCR analysis of the CCL4 and CXCR5 transcript levels in beta-coronavirus-infected HCT-8 cells also showed a robust upregulation of these genes in infected cells. It should be noted that upregulation of CXCL10, CXCL11, and IFIT2 in the nasopharyngeal samples has been proposed to accurately predict the presence of respiratory virus infection (Landry and Foxman, 2018). Lack of induction of these genes in old age infected subjects may indicate an inability of the host cells to detect viral entry. This, in combination with downregulation of genes involved in antiviral immunity, likely contributes to severe disease outcomes, which is consistent with severe COVID-19 manifestations in older patients. However, more studies using the lung biopsy samples of SARS-CoV-2-infected young and old age patients will be required to confirm these findings.

What processes may be causing/contributing to defective immune response in the SARS-CoV-2-infected cells? Autophagy- and mitochondria-related processes were two other prominent categories of the BPs that were exclusively impacted in the hCoV-infected cells.

Gene expression analysis of SARS-CoV-2-infected A549 and Calu3 cells revealed differential expression of genes involved in autophagic processes. In contrast, most of the DE genes from human nasopharyngeal sample comparison that annotated to autophagy processes were downregulated. SARS-CoV-2-infected A549 and Calu3 cell lines and human nasopharyngeal samples expressed significantly low levels of mTOR and LAMTOR genes. The regulatory associated protein of mTOR (RAPTOR) expression was also significantly decreased in all SARS-CoV-2-infected samples. mTOR inhibits autophagy (Kapuy et al., 2014; Rabanal-Ruiz and Korolchuk, 2018). Consistent with the mTOR function, the autophagy inducing microphthalamia-associated transcription factors (MiTF/TFE) and unc-51-like autophagy activating kinase 1 (ULK1) were upregulated in the infected A549 cells. Concurrent with these data, inhibition of mTORC1 in SARS-CoV-2-infected human bronchial epithelial cells NCI-H1299 and monkey kidney cells (VeroFM) has also been reported (Gassen et al., 2020). These observations suggest that downregulation of mTOR may result in autophagy induction upon SARS-CoV-2 infection in A549 cells. However, contrary to the expression profiles of infected A549 cells, the autophagy initiation and nucleation genes were downregulated in infected human nasopharyngeal cells compared to control samples. It is worth noting that a past study in mouse embryonic cells showed that coronavirus infection induced autophagy and that coronavirus mouse hepatitis virus (MHV) replication was impaired in atg5–/– cells (Prentice et al., 2004). While the gene expression data from SARS-CoV-2-infected cell lines and human samples indicate changes in autophagy gene expression, very little can be inferred about the changes to the autophagic flux, which is the capacity of the cells to degrade the autophagosome by fusing with the lysosomes. Studies have shown that dengue and enteroviral infections inhibit autophagic flux in host cells by decreasing p62 or cleaving SNAP29, respectively, to facilitate infection (Metz et al., 2015; Mohamud et al., 2018). Consistent with this observation, we found that the autophagic flux inducing p62 (SQSTM1) and SNAP29 gene expression level were down in SARS-CoV-2-positive human samples. Moreover, inhibition of S-phase kinase-associated protein 2 (SKP2) as E3 ligase decreased Beclin1 (BECN1) degradation and increased autophagic flux, which, in turn, decreased MERS-CoV (Gassen et al., 2019; Yang and Shen, 2020). However, SKP2 expression in both SARS-CoV-2-infected cell lines and human samples was significantly low. Nevertheless, drugs known to increase autophagic flux has been shown to impede SARS-CoV-2 infection (Gassen et al., 2020; Gorshkov et al., 2020). It is likely that autophagic flux is decreased in human samples by downregulation of p62 and SNAP29 and in SARS-CoV-2-infected A549 cells potentially through upregulation of glycogen synthase kinase 3 beta (GSK3B), which has been shown to impair lysosome acidification (Weikel et al., 2016). Consistently, GSK3B inhibition has been shown to increase autophagy flux in mice liver and human pancreatic cancer cells (Marchand et al., 2015; Ren et al., 2016). Additionally, several lysosome acidification genes were concordantly downregulated in both SARS-CoV-2-infected A549 cells and human samples. Since impaired lysosome acidification has been associated with impaired autophagic flux (Yim and Mizushima, 2020), drugs targeting lysosome reacidification or increasing autophagic flux could potentially be tested as a therapeutic intervention to SARS-CoV-2 infection. Another independent study also showed that beta-coronavirus deacidifies lysosomes as a mechanism to facilitate viral infection and egress from the host cells (Ghosh et al., 2020). Together, these findings suggest that autophagy and regulation of autophagic flux may be central to SARS-CoV-2 infection and that their differential regulation may be key to the control of the infection.

In addition to changes in expression of genes involved in autophagic processes, several genes involved in the mitochondrial processes were downregulated in SARS-CoV-2-infected A549 cells and human samples. This is consistent with our current understanding that viruses either induce or inhibit various mitochondrial processes as part of their replication and dissemination efforts (Anand and Tikoo, 2013). Infection of cells with SARS-CoV-2 at higher viral titer downregulated the genes in the mitochondrial processes. Consistently, WGCNA of SARS-CoV-2-infected A549 datasets also identified a DE gene cluster that annotated to the mitochondrial organization and translation processes. Subsequent GeneMANIA analysis identified a PPI subnetwork of genes involved in mitochondrial translation, which were coordinately downregulated in SARS-CoV-2-infected cells. Since mitochondrial import and translation are interlinked (Mokranjac and Neupert, 2010; Sanchez-Caballero et al., 2016), we found that several mitochondrial complex I and translocase genes were downregulated in the SARS-CoV-2-infected cells. Given the extensive crosstalk between autophagy and mitochondrial function (Graef and Nunnari, 2011; Rambold and Lippincott-Schwartz, 2011), it is likely that perturbations in autophagy and mitochondrial processes observed in SARS-CoV-2-infected cells are interlinked. It is worth noting that the mTORC1 complex, which comprises the mTOR and RAPTOR, stimulates synthesis of mitochondrial ribosomal, complex I proteins (Morita et al., 2013, 2015) and mitochondrial fission process 1 (MTFP1) (Morita et al., 2017). Consistently, we found decreased expression of mitochondrial ribosomal and complex I genes, which is likely a result of decreased mTOR and RAPTOR expression in SARS-CoV-2-infected cells. Additionally, decreased expression of MTFP1 that may impede mitochondrial fission resulting in hyperfused mitochondria (Tondera et al., 2005) was specifically observed in SARS-CoV-2-infected A549 cells. While MTFP1 gene expression was undetectable in human nasopharyngeal samples, another mitochondrial fission promoting SOCS6 (Lin et al., 2013) was downregulated in these infected samples. Past studies have shown that hyperfused or elongated mitochondria in dengue- and SARS-infected cells can suppress interferon signaling and innate immunity (Shi et al., 2014; Barbier et al., 2017; Das and Chakrabarti, 2020). Furthermore, reduced complex I expression has been found in many cancer cells and is shown to affect the oxidative phosphorylation, which also impacts the immune cell function (Simonnet et al., 2003; Bonora et al., 2006; Baracca et al., 2010; Won et al., 2015; Angajala et al., 2018). Together, these data highlight that SARS-CoV-2-infected cells have decreased mTOR expression and perturbation in autophagy and mitochondrial processes, which, in turn, could properly impede the immune response to infection. Further studies will, however, be required to delineate which of these perturbations are the direct result of SARS-CoV-2 infection and/or contribute to pathogenesis/severe clinical manifestations.

## Summary

In summary, we have presented a detailed DE and coexpression network analysis of the RNA-seq data from SARS-CoV-2-infected A549 cells. Using the gene expression profiles of A549 and Calu3 cells infected with IAV or MERS-CoV, we concluded that perturbations in cytokine signaling and inflammation processes, downregulation of genes in the mitochondrial processes, and perturbation of autophagy were uniquely observed in novel coronavirus-infected cells. To validate the findings from the cell line data, gene expression analysis of control and SARS-CoV-2-positive human nasopharyngeal samples was performed. Consistent with the cell line data, DE genes from human data also significantly annotated to inflammation, autophagy, and mitochondrial processes. It is likely that perturbation of autophagy and mitochondrial processes may impede an effective immune response leading to severe outcomes. Furthermore, age-stratified human nasopharyngeal was used to analyze gene expression changes in control vs. high viral load positive old age (>60 years) samples. This analysis also revealed several BPs that were concordantly impacted in both cell line and human datasets, with few differences. Specifically, genes encoding mTOR were downregulated in infected cells, which likely caused downregulation of mitochondrial ribosomal genes in both cell line and human datasets. Additionally, genes encoding mitochondrial complex I and lysosome acidification were also concurrently downregulated in infected cells from both datasets. Several inflammation process genes and autophagy genes were discordantly regulated in both datasets. It is likely that the autophagic flux is impeded in infected cells lines and human samples due to increased expression of GSK3B gene or downregulation of p62 and SNAP29, respectively, which may further promote viral propagation. These data suggest that drugs that enhance autophagic flux or increase lysosome acidification could be tested as intervention strategies. Increased expression of inflammation process genes in A549 cells is likely due to these cells representing a severe COVID-19 infection state. Consistently, we found upregulation of inflammatory process genes in expression profiles of the COVID-19 lung biopsy sample. These data support a central role for cytokine/inflammation processes in COVID-19 pathogenesis. Finally, using the age-stratified expression profiles of infected human nasopharyngeal samples, we identified muted or downregulation of several chemokines, ISGs, and tripartite motif genes that are critical for innate immunity and antiviral signaling. It is likely that defective antiviral response in old age patients in combination with perturbations in autophagy and mitochondrial processes could result in severe COVID-19 disease state often seen in older population. In summary, using gene expression data of SARS-CoV-2-infected cells, we show that viral infection of host cells results in perturbations in specific aspects of autophagy and mitochondrial processes. Future studies focusing on how these perturbations either contribute to viral propagation or impede an effective immune response will be required to gain more understanding of the viral pathogenesis.

## Materials and Methods

### Data Collection

Raw gene count matrix for bulk RNA-seq was obtained from GEO (accession number GSE147507) (Blanco-Melo et al., 2020). The data contained gene expression count matrix of two lung carcinoma cell lines A549 (Lieber et al., 1976; Holownia et al., 2016) and Calu3 (Shen et al., 1994; Martens et al., 2018). In this dataset, the A549 treatment conditions included mock, infection with IAV (*N* = 2 per group), and infection with SARS-CoV-2 at 2 (high titer, *N* = 3 per group) and 0.2 (low titer, *N* = 3 per group) multiplicity of infection (MOI), after transduction with a vector expressing human ACE2 (hACE2) (*N* = 3 per group). The adenovirus Ad-GFP-h-ACE2 from Vector Biolabs was used for transduction of A549 cells at 500 MOI (Blanco-Melo et al., 2020). Subsequently, cells were infected with SARS-CoV-2 [Isolate USA-WA1/2020 (NR-52281)] at 0.2 or 2 MOI as indicated (Blanco-Melo et al., 2020). From this dataset, the raw gene count matrix for two healthy human lung biopsy samples and one COVID-19 sample (two technical replicates) and Calu3 cells infected with SARS-CoV-2 at 2 MOI (*N* = 3 per group) were also analyzed. Since the A549 cells infected with SARS-CoV-2 at high and low MOI were used in the network analysis, and DE genes from A549 and Calu3 comparisons were used, a boxplot of the normalized counts showed that all samples were comparable with no outlier (data not shown). Additionally, gene expression data in FPKM were downloaded from GEO (accession number GSE139516) (Zhang et al., 2020) for the Calu3 cell line infected with MERS-CoV and mock (*N* = 3 per group). Differential expression analysis was performed on mock- and MERS-CoV-infected cells for 24 h. Human lung single-cell RNA-seq (scRNA-seq) data with 57 annotated cell types were downloaded from Synapse (accession syn21041850) (Travaglini et al., 2020). For validation study, human nasopharyngeal gene expression matrix was obtained from GEO (accession number GSE152075) (Lieberman et al., 2020). Using patient age and viral load information, the samples were further classified into young (<40 years) and old age (>60 years) that were either negative or positive with low or high viral loads.

### Data Analysis

#### RNA-Seq Analysis and Network Analysis

Differential expression analysis was performed using limma-voom and limma trends (Law et al., 2014; Ritchie et al., 2015). Genes with adjusted *p* < 0.05 were considered DE. The DE genes were tested for pathway enrichment using clusterProfiler and pathways with *q-*values (i.e., *p*-values corrected for multiple comparison) < 0.05 were considered significant (Yu et al., 2012). To perform the consensus WGCNA (Langfelder and Horvath, 2008), first the pooled control and hACE2-transduced SARS-CoV-2-infected A549 cells at low and high titer expression data were batch corrected using combat (from the sva R package) (Leek et al., 2012), and the low expressing genes with count < 5 in four out of six samples were removed. The batch-corrected normalized count was analyzed using the WGCNA R package (Langfelder and Horvath, 2008) with default parameters. A total of 50 coexpressing modules were identified, of which DE genes in correlated modules > 50 genes in size were selected for downstream analysis. For subnetwork analysis, GeneMANIA database (Mostafavi et al., 2008) was used to identify potential PPI between the DE genes from the correlated modules. The PPI networks were then overlaid with the fold-change information using Cytoscape (Shannon et al., 2003). Prior to DE analysis of the human nasopharyngeal gene expression data (Lieberman et al., 2020), the gene count matrix was subset to only include the SARS-CoV-2-positive and -negative samples that were either <40 years (young) or >60 years (old) and exclude samples that were positive with medium load viral titer. Using the sva R package and batch information, the count matrix was batch corrected across three experimental groups: control (or negative), low viral load, and high viral load in both young and old groups. DE analysis of this dataset was performed as described above. DE analysis was also performed on batch-corrected negative (control) vs. SARS-CoV-2-positive human nasopharyngeal samples.

#### Code Availability

The differential gene expression and pathway enrichment results for all the comparisons are included in the supplement. All the R codes used to analyze the data and make figures can be found at https://github.com/NHLBI-BCB/COVID-19_Transcriptomics. There is no restriction on data access.

#### Generating Pathway Enrichment Summary Map

The pathway enrichment summary map was generated using the indicated pathway enrichment results presented in Supplementary Table 4. To compare the pathway enrichment results from two different comparisons, the “Description,” “q-value,” and “GeneID” information from each pathway enrichment table were used to compute similarity of the pathways and overlap of genes between the pathways. Using this information, the enrichment Map (Merico et al., 2010) app in Cytoscape creates a graphical network, where the nodes represent a pathway. If the genes annotated to pathway is shared with another pathway (which arises due to genes usually getting annotated to more than one pathway), an edge is drawn between the pathway nodes to show this information. Additionally, if DE genes from two different comparisons were significantly annotated to the same pathway/node, that node is highlighted with two colors. The common terms in the pathway description were then used to annotate/label a group of nodes using the Enrichment Map (Merico et al., 2010) and AutoAnnotate (Kucera et al., 2016) apps in Cytoscape. R (Foundation for Statistical Computing, Vienna, Austria. URL https://www.R-project.org/) was used for data visualization. The datasets supporting the results of this article are also available from Figshare (https://doi.org/10.6084/m9.figshare.12272351.v8) (Mehdi, 2020).

#### Single-Cell RNA-Seq Analysis

Analysis of human lung single-cell RNA-seq (scRNA-seq) data with 57 annotated cell types was performed in R (v3.6) using Seurat (v3.1.1) (Stuart et al., 2019). The UMI (Unique Molecular Identifier) count matrix was filtered for genes expressed in < 3 cells and normalized using *SCTransform* implemented in Seurat. DE genes were computed for 57 cell types using *FindAllMarkers* implemented in Seurat with default parameters. The UMAP plot was plotted using the top 50 principal components computed from the expression of highly variable genes selected by *SCTransform*.

#### Quantitative Real-Time Polymerase Chain Reaction (qRT-PCR) Analysis

HCT-8 cell line (CCL-244) and the RNA of HCT-8 cells (VR-1558D) infected with OC43, a beta-coronavirus 1 strain, were purchased from ATCC. The control HCT-8 RNA was isolated after culturing the cells in RPMI medium supplemented with 10% fetal bovine serum (Gibco). The total RNA was isolated using the miRNeasy isolation Kit (Qiagen). The cDNA was synthesized using SuperScriptIII (Invitrogen). The transcripts were measured using FastStart Essential DNA Green Master (Roche). The 18s transcript measurements were used as control. All the primers were ordered from Qiagen QuantiTect assay. Four technical replicates are reported. *p*-values from Student's *t*-test are reported.

## Data Availability Statement

The original contributions presented in the study are included in the article/Supplementary Material, further inquiries can be directed to the corresponding author/s.

## Author Contributions

KS and MP conceived the project. KS, YC, and MP designed the workflow and performed the analyses. KS, FS, and MP collected the data. KS, SH, KH, and MNS validated the results. KS and JJ drafted the manuscript. All authors contributed to the analysis and evaluation of the results. All authors contributed to the article and approved the submitted version.

## Conflict of Interest

The authors declare that the research was conducted in the absence of any commercial or financial relationships that could be construed as a potential conflict of interest.

## Supplementary Material

Supplementary Figure 1 **(A)** Volcano plot showing DE genes that were up (red color dots) and down regulated (blue color dots) in hACE2 transduced A549 cells infected with SARS-CoV-2 (high MOI). **(B)** Top 25 pathways from the pathway enrichment analysis of the DE genes from the mock vs. SARSCoV-2 (high MOI) comparison is presented as a horizontal bar plot, where x axis represents the log10 transformed q-value and the color of the horizontal bar is scaled blue to red representing low to high q-values, respectively. **(C)** Volcano plot showing DE genes that were up (red color dots) and down regulated (blue color dots) in SARS-CoV-2 (low MOI) infected A549 cells that were transduced with hACE2. **(D)** Top 25 pathways from the pathway enrichment analysis of the DE genes from the mock vs. SARS-CoV-2 (low MOI) comparison is presented as a horizontal bar plot, where x axis represents the –log10 transformed q-value and the color of the horizontal bar is scaled blue to red representing low to high q-values, respectively. DE, differentially expressed; MOI, multiplicity of infection. **(E)** Plot showing correlation between marker genes from different lung subpopulations (on x-axis) and hACE2 transduced A549 and Calu3 cells lines (color coded independent samples with legend at the bottom of the plot).

Supplementary Figure 2 **(A)** Correlation plot between mean gene expression from SARS-CoV-2 infected hACE2 transduced A549 (x axis) and Calu3 (y axis) cells. **(B)** Venn diagram showing overlap between DE genes from mock vs. SARS-CoV-2 A549 and Calu3 cell comparisons. **(C)** Pathway enrichment summary map for mock vs. SARS-CoV-2 comparisons in Calu3 (blue nodes) and hACE2 transduced A549 (red nodes) cells. Each node represents a pathway/biological process (BP). The node size is proportional to the number of DE genes overlapping with the BP. The nodes that share genes are connected with edges. The black circle outlines group the gene ontology (GO) terms of similar BPs. Single color nodes are pathways that are distinctly enriched by DE genes from one comparison. Two colored nodes are pathways enriched by DE genes from both comparisons. The DE genes from both comparisons enriched in inflammation, ROS, mitochondria, and autophagy processes.

Supplementary Figure 3 **(A)** Volcano plot showing DE genes that were up (red color dots) and down regulated (blue color dots) in IAV infected A549 cells. **(B)** Top 25 pathways from the pathway enrichment analysis of the DE genes from the mock vs. IAV comparison is presented as a horizontal bar plot, where x axis represents the –log10 transformed q-value and the color of the horizontal bar is scaled blue to red representing low to high q-values, respectively. **(C)** Venn diagram showing DE genes overlap between mock vs. SARS-CoV-2 (High MOI) and mock vs. IAV comparisons. DE, differentially expressed; MOI, multiplicity of infection.

Supplementary Figure 4 **(A)** Volcano plot showing DE genes that were up (red color dots) and down regulated (blue color dots) in SARS-CoV-2 infected Calu3 cells. **(B)** Top 25 pathways from the pathway enrichment analysis of the DE genes from the mock vs. SARS-CoV-2 comparison in Calu3 is presented as a horizontal bar plot, where x axis represents the –log10 transformed q-value and the color of the horizontal bar is scaled blue to red representing low to high q-values, respectively.

Supplementary Figure 5 **(A)** Top 25 pathways from the pathway enrichment analysis of the DE genes from the positive (infected) vs. negative human nasopharyngeal samples comparison is presented as a horizontal bar plot, where x axis represents the –log10 transformed q-value and the color of the horizontal bar is scaled blue to red representing low to high q-values, respectively. **(B)** Venn diagram showing DE genes overlap between control vs. high viral load old age human samples and mock vs. SARS-CoV-2 infected A549 cells comparisons. **(C)** Pathway enrichment result of common DE genes indicates in figure **(B)**, that were concordantly downregulated in both datasets is presented as a horizontal bar plot, where x axis represents the –log10 transformed q-value and the color of the horizontal bar is scaled blue to red representing low to high q-values, respectively. DE, differentially expressed.

Supplementary Figure 6 Heatmap of the mean expression values of the indicated genes in young and old human samples that were negative (control) or positive with either high or low viral loads of SARS-CoV-2 virus is presents. **(A)** Heatmap of interferon signaling genes. **(B)** Heatmap of mitochondrial ribosomal genes. **(C)** Heatmap of mitochondrial complex I genes. **(D)** Heatmap of lysosome acidification genes. The orange and blue color bands represent upregulated and downregulated genes, respectively.

Supplementary Figure 7 DE genes from COVID-19 lung compared to healthy lungs show robust upregulation of immunity, cytokines, and inflammatory processes. **(A)** Volcano plot showing DE genes that were up (red color dots) and down regulated (blue color dots) in COVID-19 lung biopsy samples compared to healthy samples. **(B)** Pathway enrichment summary map for healthy vs. COVID-19 lungs (technical replicates) (blue nodes). Each node represents a pathway/biological process (BP). The node size is proportional to the number of DE genes overlapping with the BP. The nodes that share genes are connected with edges. The black circle summarizes the gene ontology (GO) terms of similar BPs. The DE genes from healthy vs. COVID-19 lung comparison predominantly enriched in inflammation and immunity related processes. **(C)** Pathway enrichment result of DE genes upregulated in COVID-19 lung vs. healthy lung biopsy samples is presented as a horizontal bar plot, where x axis represents the –log10 transformed q-value and the color of the horizontal bar is scaled blue to red representing low to high q-values, respectively. **(D)** Pathway enrichment result of DE genes downregulated in COVID-19 lung vs. healthy lung biopsy samples is presented as a horizontal bar plot, where x axis represents the log10 transformed q-value and the color of the horizontal bar is scaled blue to red representing low to high q-values, respectively.

Supplementary Table 1 Consensus module name and size (gene numbers) and its overlap with significant genes.

Supplementary Table 2 List of marker genes of lung cell types from single cell RNA-seq data.

Supplementary Table 3 Gene Differential Expression (GDE) Analysis results in COVID-19 transcriptomics comparisons.

Supplementary Table 4 Pathway Enrichment analysis results of GDE genes in COVID-19 transcriptomics comparisons.
