# Supplementary figures and images for: Network Analysis and Transcriptome Profiling Identify Autophagic and Mitochondrial Dysfunctions in SARS-CoV-2 Infection
Source: Front Genet. 2021 Mar 16;12:599261. doi: 10.3389/fgene.2021.599261 (PMC8008150; doi:10.3389/fgene.2021.599261)

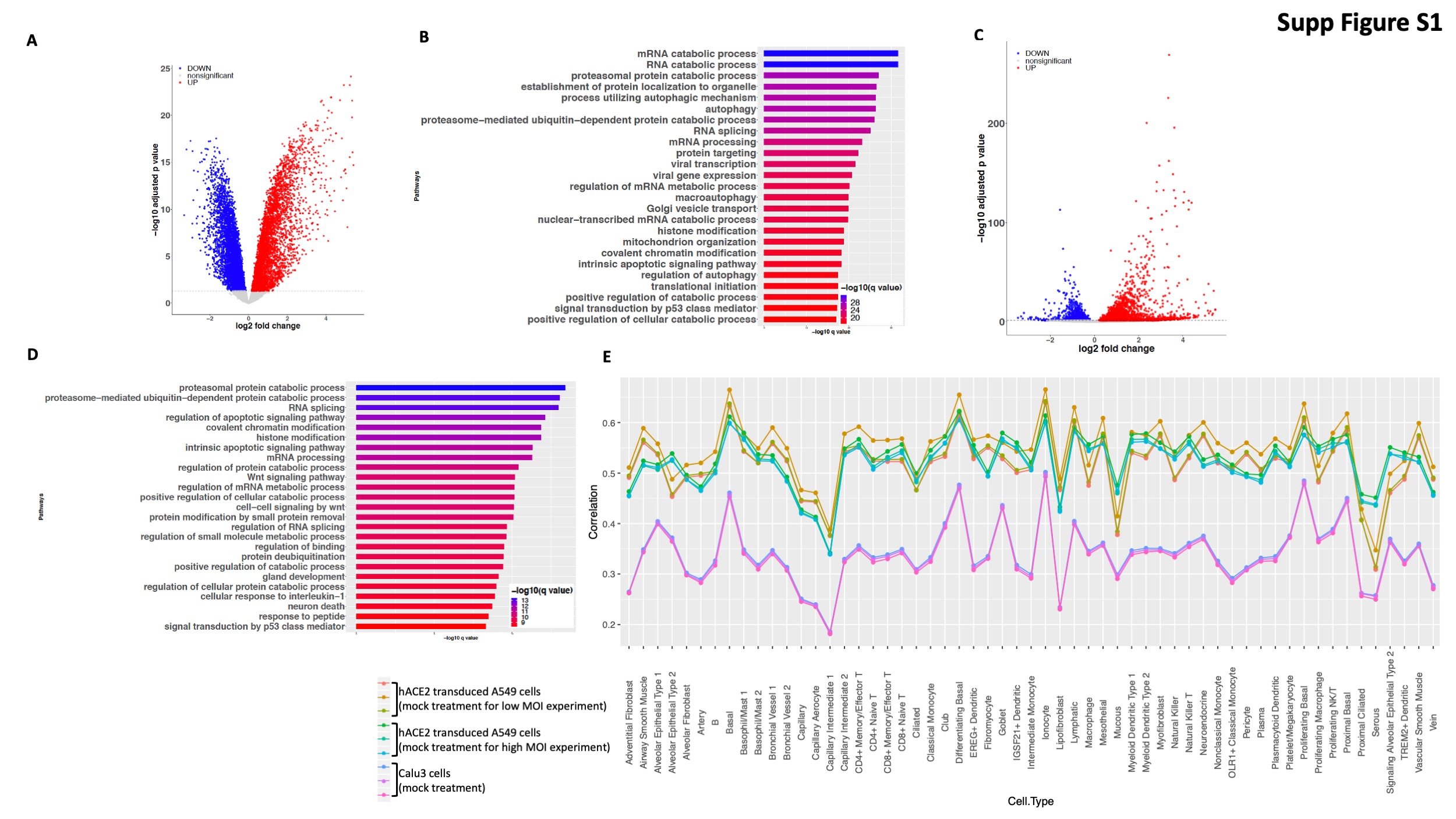

Supplement: Supplementary Figure 1 — (A) Volcano plot showing DE genes that were up (red color dots) and down regulated (blue color dots) in hACE2 transduced A549 cells infected with SARS-CoV-2 (high MOI). (B) Top 25 pathways from the pathway enrichment analysis of the DE genes from the mock vs. SARSCoV-2 (high MOI) comparison is presented as a horizontal bar plot, where x axis represents the log10 transformed q-value and the color of the horizontal bar is scaled blue to red representing low to high q-values, respectively. (C) Volcano plot showing DE genes that were up (red color dots) and down regulated (blue color dots) in SARS-CoV-2 (low MOI) infected A549 cells that were transduced with hACE2. (D) Top 25 pathways from the pathway enrichment analysis of the DE genes from the mock vs. SARS-CoV-2 (low MOI) comparison is presented as a horizontal bar plot, where x axis represents the –log10 transformed q-value and the color of the horizontal bar is scaled blue to red representing low to high q-values, respectively. DE, differentially expressed; MOI, multiplicity of infection. (E) Plot showing correlation between marker genes from different lung subpopulations (on x-axis) and hACE2 transduced A549 and Calu3 cells lines (color coded independent samples with legend at the bottom of the plot). [file Image_1.JPEG]

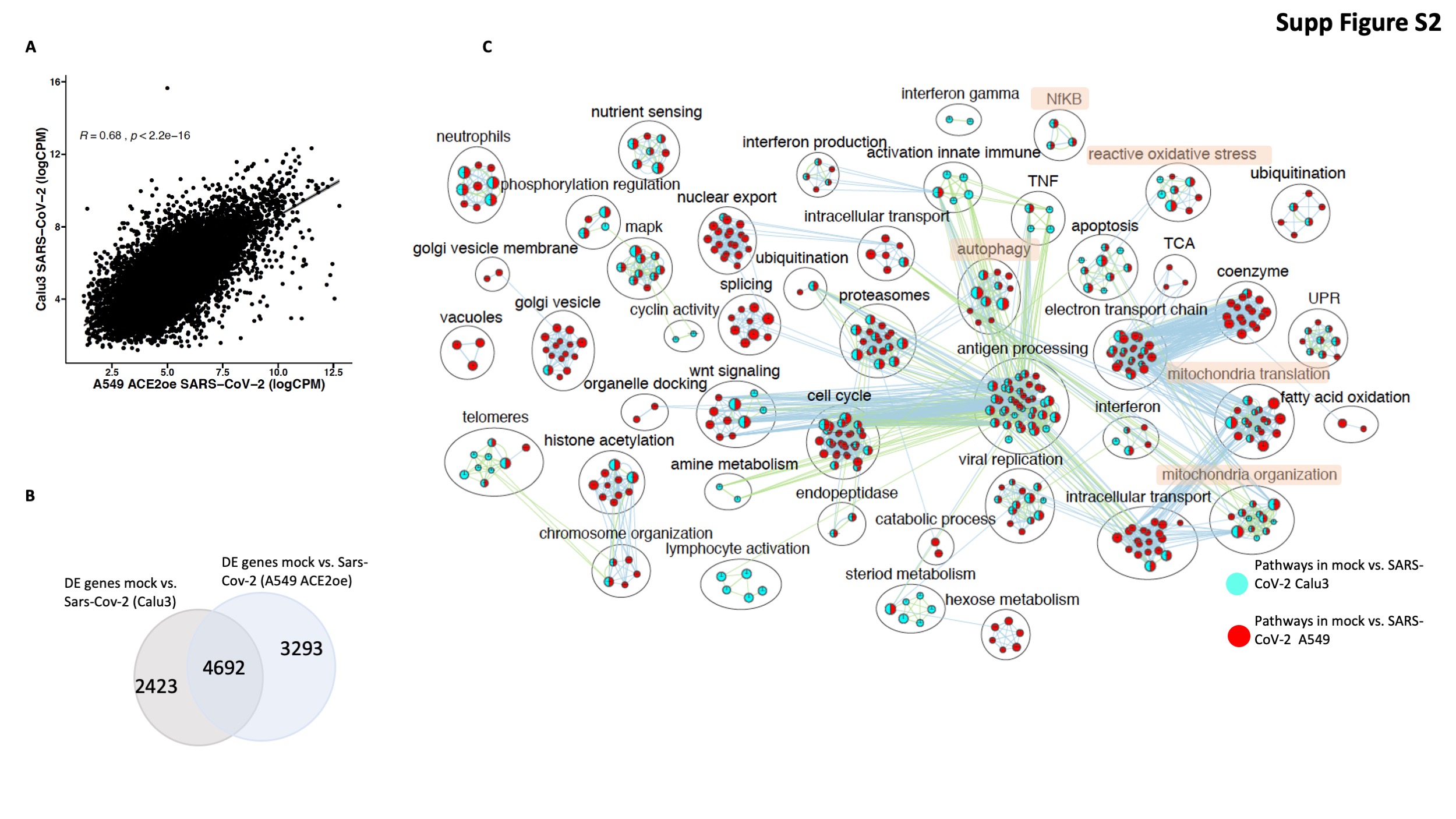

Supplement: Supplementary Figure 2 — (A) Correlation plot between mean gene expression from SARS-CoV-2 infected hACE2 transduced A549 (x axis) and Calu3 (y axis) cells. (B) Venn diagram showing overlap between DE genes from mock vs. SARS-CoV-2 A549 and Calu3 cell comparisons. (C) Pathway enrichment summary map for mock vs. SARS-CoV-2 comparisons in Calu3 (blue nodes) and hACE2 transduced A549 (red nodes) cells. Each node represents a pathway/biological process (BP). The node size is proportional to the number of DE genes overlapping with the BP. The nodes that share genes are connected with edges. The black circle outlines group the gene ontology (GO) terms of similar BPs. Single color nodes are pathways that are distinctly enriched by DE genes from one comparison. Two colored nodes are pathways enriched by DE genes from both comparisons. The DE genes from both comparisons enriched in inflammation, ROS, mitochondria, and autophagy processes. [file Image_2.JPEG]

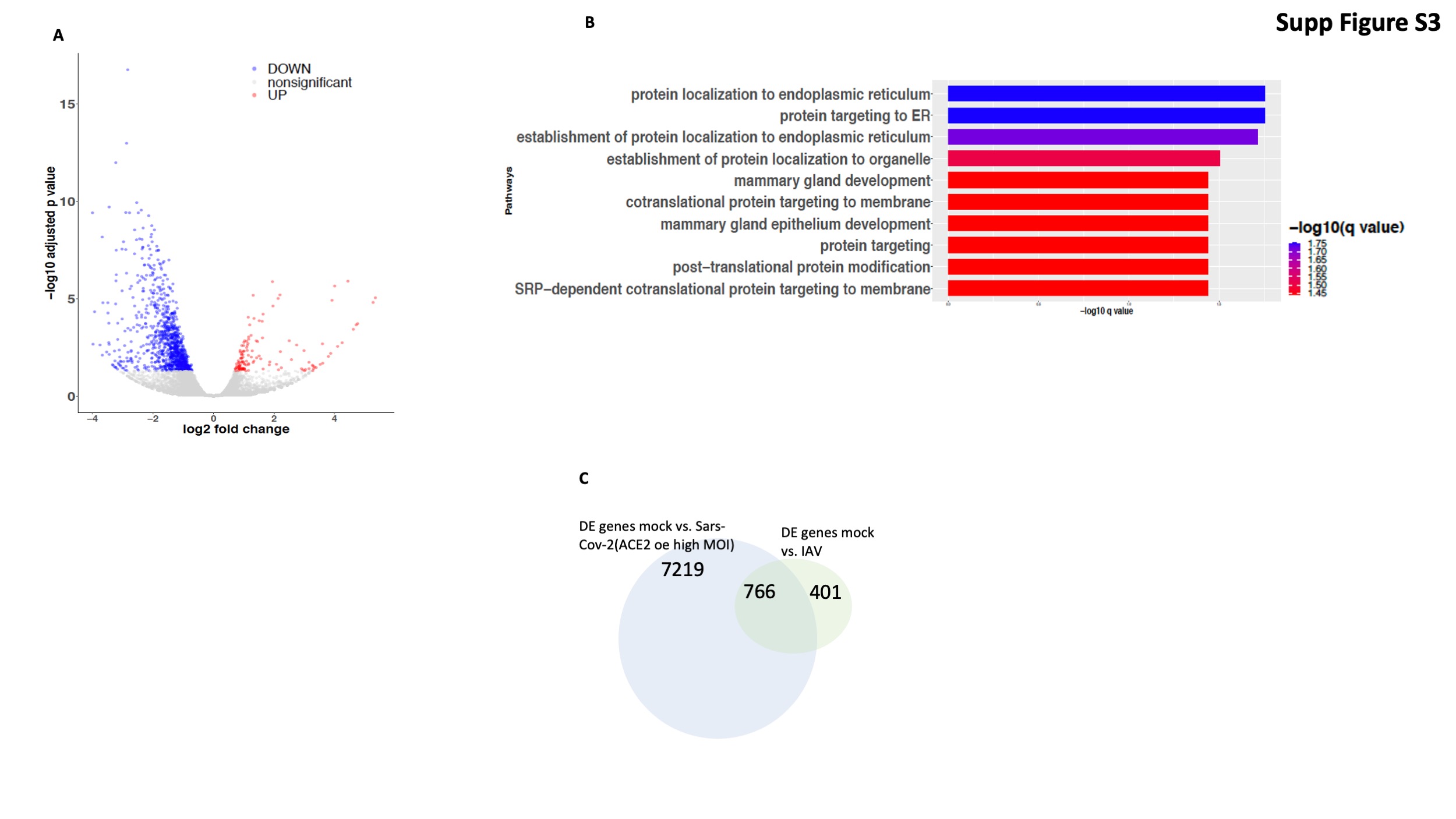

Supplement: Supplementary Figure 3 — (A) Volcano plot showing DE genes that were up (red color dots) and down regulated (blue color dots) in IAV infected A549 cells. (B) Top 25 pathways from the pathway enrichment analysis of the DE genes from the mock vs. IAV comparison is presented as a horizontal bar plot, where x axis represents the –log10 transformed q-value and the color of the horizontal bar is scaled blue to red representing low to high q-values, respectively. (C) Venn diagram showing DE genes overlap between mock vs. SARS-CoV-2 (High MOI) and mock vs. IAV comparisons. DE, differentially expressed; MOI, multiplicity of infection. [file Image_3.JPEG]

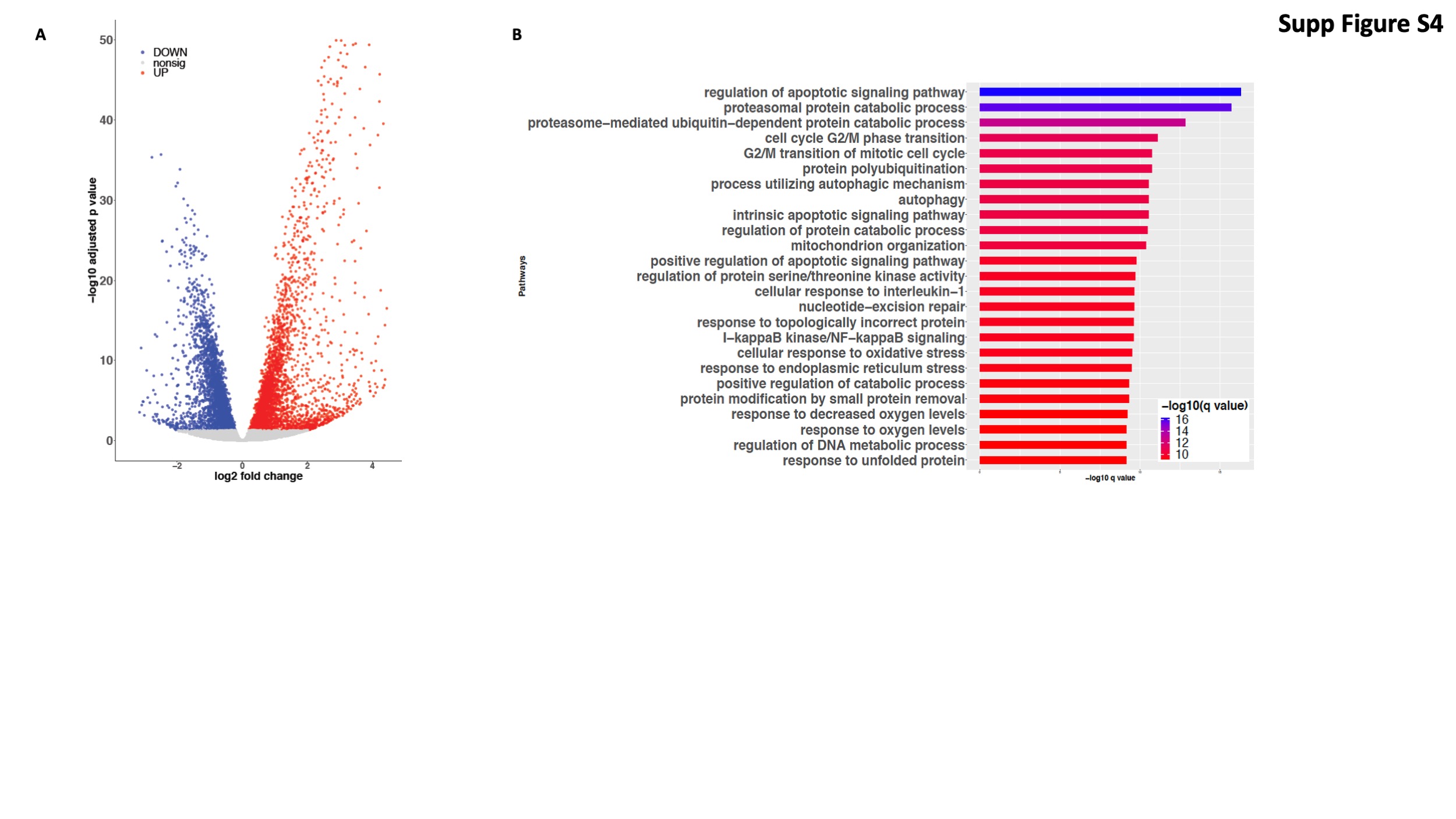

Supplement: Supplementary Figure 4 — (A) Volcano plot showing DE genes that were up (red color dots) and down regulated (blue color dots) in SARS-CoV-2 infected Calu3 cells. (B) Top 25 pathways from the pathway enrichment analysis of the DE genes from the mock vs. SARS-CoV-2 comparison in Calu3 is presented as a horizontal bar plot, where x axis represents the –log10 transformed q-value and the color of the horizontal bar is scaled blue to red representing low to high q-values, respectively. [file Image_4.JPEG]

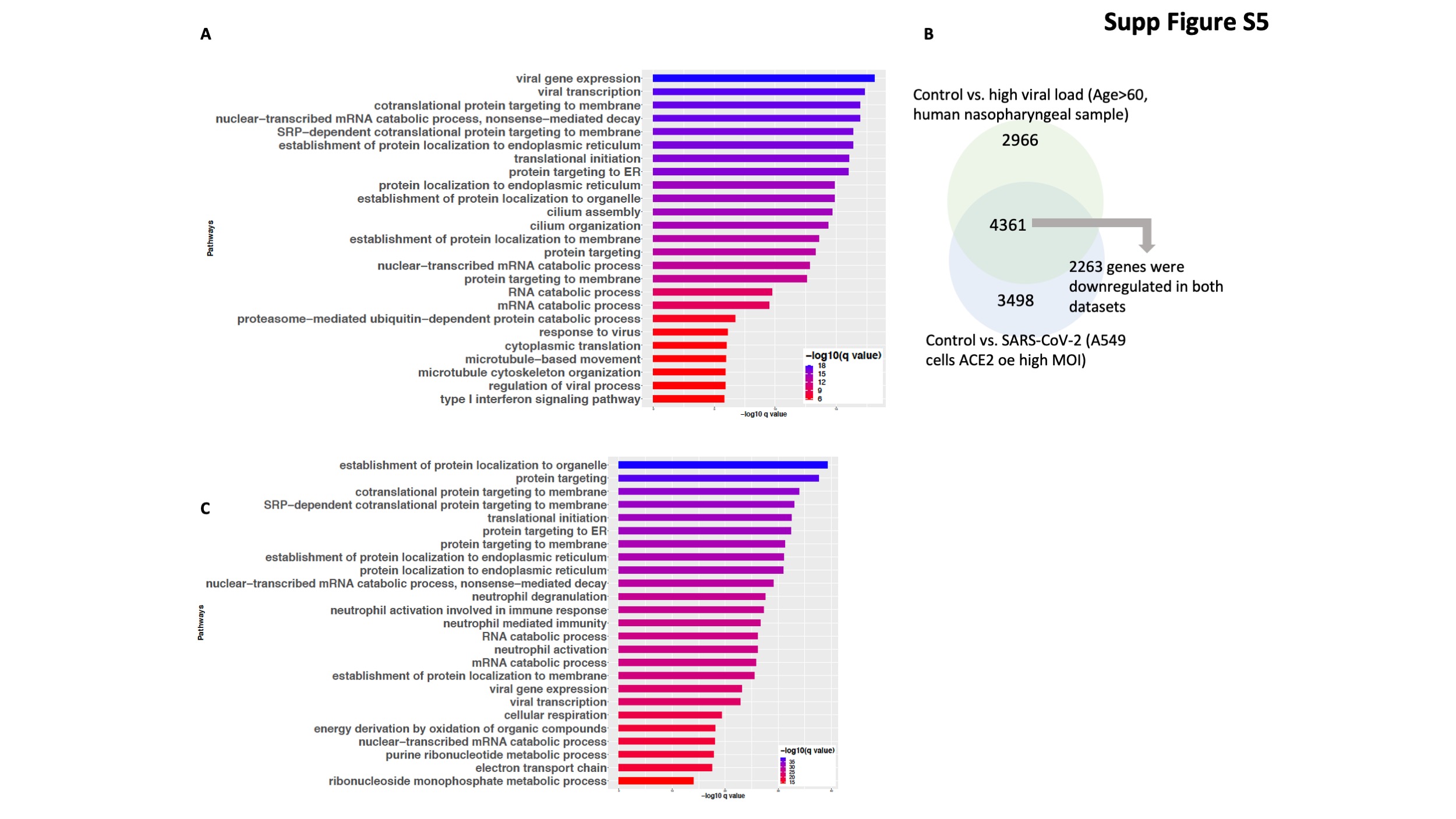

Supplement: Supplementary Figure 5 — (A) Top 25 pathways from the pathway enrichment analysis of the DE genes from the positive (infected) vs. negative human nasopharyngeal samples comparison is presented as a horizontal bar plot, where x axis represents the –log10 transformed q-value and the color of the horizontal bar is scaled blue to red representing low to high q-values, respectively. (B) Venn diagram showing DE genes overlap between control vs. high viral load old age human samples and mock vs. SARS-CoV-2 infected A549 cells comparisons. (C) Pathway enrichment result of common DE genes indicates in figure (B), that were concordantly downregulated in both datasets is presented as a horizontal bar plot, where x axis represents the –log10 transformed q-value and the color of the horizontal bar is scaled blue to red representing low to high q-values, respectively. DE, differentially expressed. [file Image_5.JPEG]

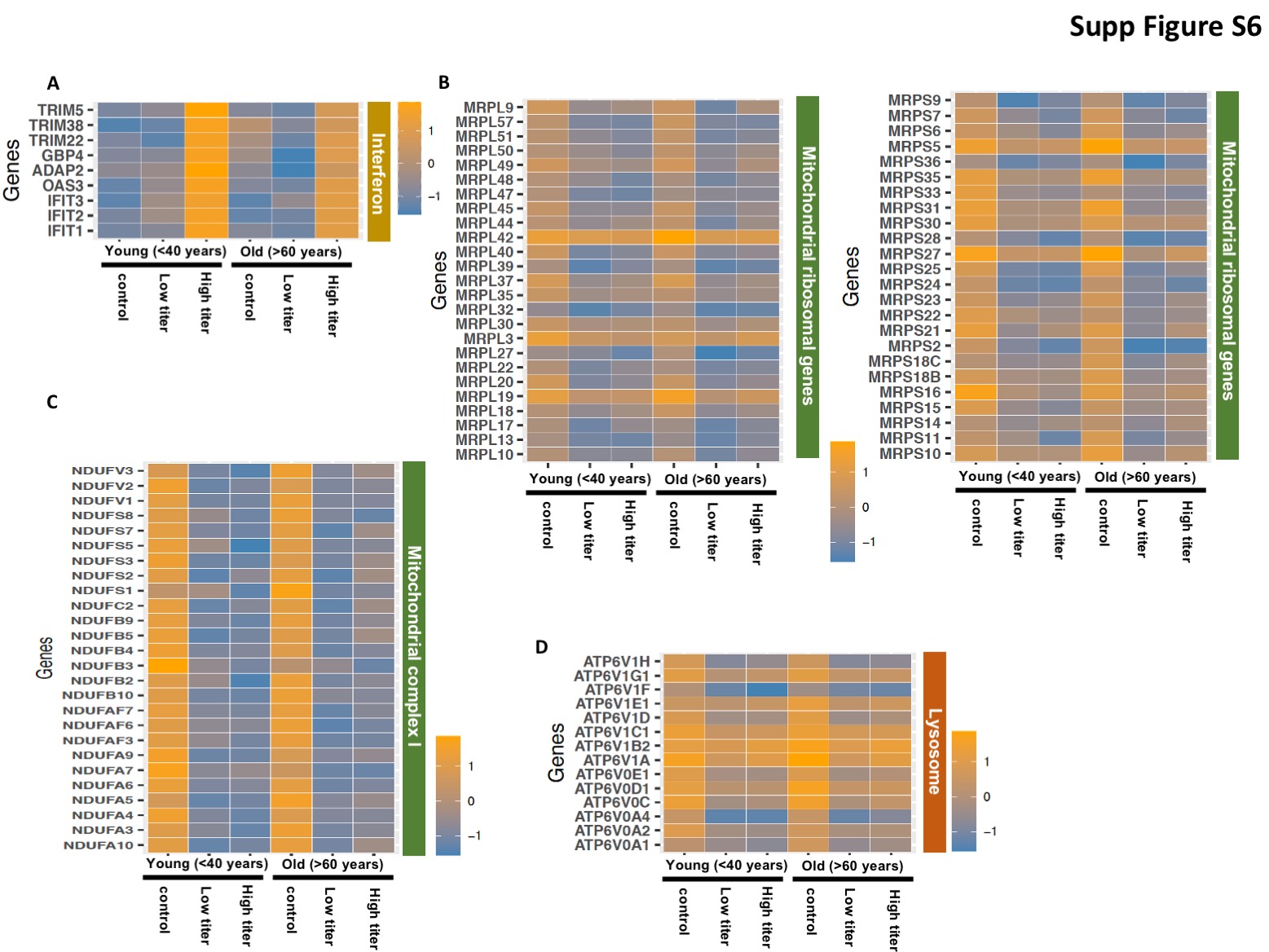

Supplement: Supplementary Figure 6 — Heatmap of the mean expression values of the indicated genes in young and old human samples that were negative (control) or positive with either high or low viral loads of SARS-CoV-2 virus is presents. (A) Heatmap of interferon signaling genes. (B) Heatmap of mitochondrial ribosomal genes. (C) Heatmap of mitochondrial complex I genes. (D) Heatmap of lysosome acidification genes. The orange and blue color bands represent upregulated and downregulated genes, respectively. [file Image_6.JPEG]

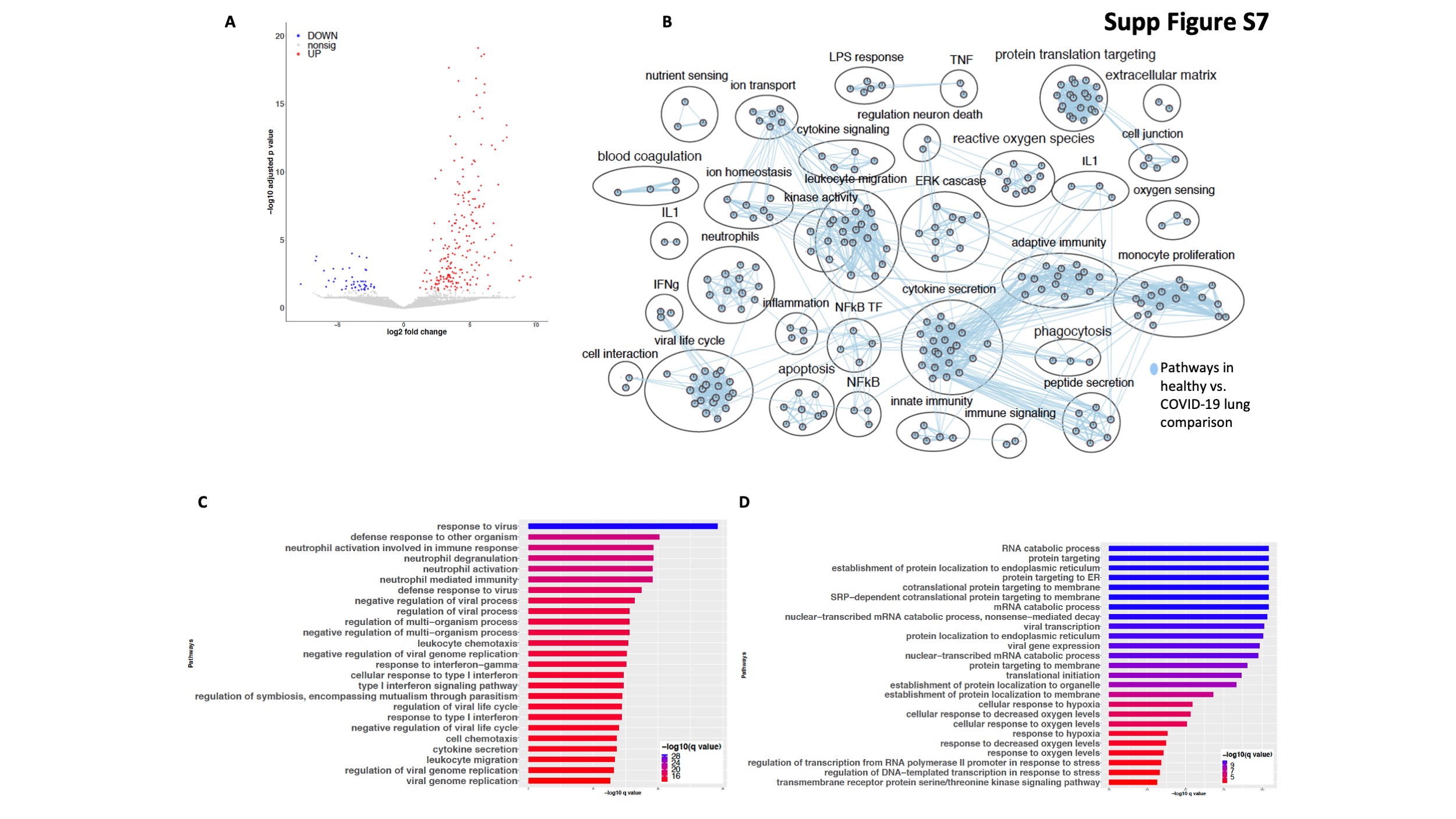

Supplement: Supplementary Figure 7 — DE genes from COVID-19 lung compared to healthy lungs show robust upregulation of immunity, cytokines, and inflammatory processes. (A) Volcano plot showing DE genes that were up (red color dots) and down regulated (blue color dots) in COVID-19 lung biopsy samples compared to healthy samples. (B) Pathway enrichment summary map for healthy vs. COVID-19 lungs (technical replicates) (blue nodes). Each node represents a pathway/biological process (BP). The node size is proportional to the number of DE genes overlapping with the BP. The nodes that share genes are connected with edges. The black circle summarizes the gene ontology (GO) terms of similar BPs. The DE genes from healthy vs. COVID-19 lung comparison predominantly enriched in inflammation and immunity related processes. (C) Pathway enrichment result of DE genes upregulated in COVID-19 lung vs. healthy lung biopsy samples is presented as a horizontal bar plot, where x axis represents the –log10 transformed q-value and the color of the horizontal bar is scaled blue to red representing low to high q-values, respectively. (D) Pathway enrichment result of DE genes downregulated in COVID-19 lung vs. healthy lung biopsy samples is presented as a horizontal bar plot, where x axis represents the log10 transformed q-value and the color of the horizontal bar is scaled blue to red representing low to high q-values, respectively. [file Image_7.JPEG]
